# Comparative Efficacy of Pharmacological and Nonpharmacological Interventions for Acne Vulgaris: A Network Meta-Analysis

**DOI:** 10.3389/fphar.2020.592075

**Published:** 2020-11-26

**Authors:** Qingyang Shi, Lizi Tan, Zhe Chen, Long Ge, Xiaoyan Zhang, Fengwen Yang, Chunxiang Liu, Junhua Zhang

**Affiliations:** ^1^ Evidence-based Medicine Center, Tianjin University of Traditional Chinese Medicine, Tianjin, China; ^2^ Evidence Based Social Science Research Center, School of Public Health, Lanzhou University, Lanzhou, China; ^3^ Department of Dermatology and Venereology, Tianjin Medical University, Tianjin, China

**Keywords:** acne vulgaris, network meta-analysis, nonpharmacological interventions, lesions reduction, adverse effects

## Abstract

Acne has several effects on physical symptoms, but the main impacts are on the quality of life, which can be improved by treatment. There are several acne treatments but less evidence comparing their relative efficacy. Thus, we assessed the comparative efficacy of pharmacological and nonpharmacological interventions for acne. We searched PubMed, Embase, and the Cochrane Central Register of Controlled Trials from inception to April 2019, to include randomized controlled trials for acne that compared topical antibiotics (TA), benzoyl peroxide (BPO), topical retinoids (TR), oral antibiotics (OA), lasers, light devices including LED device (LED), photodynamic therapy (PDT), and intense pulsed light, chemical peels (CP), miscellaneous therapies or complementary and alternative medicine (MTCAM), or their combinations. We performed Bayesian network meta-analysis with random effects for all treatments compared with placebo and each other. Mean differences (MDs) of lesions count and risk ratios of adverse events with their 95% credible intervals (CrIs) were calculated, and all interventions were ranked by the Surface Under the Cumulative Ranking (SUCRA) values. Additional frequentist additive network meta-analysis was performed to detect the robustness of results and potential interaction effects. Sensitivity analyses were carried out with different priors, and metaregression was to adjust for nine potential effect modifiers. In the result, seventy-three randomized controlled trials (27,745 patients with mild to moderate acne), comparing 30 grouped intervention categories, were included with low to moderate risk of bias. For adverse effects, OA had more risk in combination treatment with others. For noninflammatory lesions reduction, seventeen interventions had significant differences comparing with placebo and three interventions (TR+BPO: MD = −21.89, 95%CrI [−28.97, −14.76]; TR+BPO+MTCAM: −22.48 [−34.13, −10.70]; TA+BPO+CP: −20.63 [−33.97, −7.13]) were superior to others with 94, 94, and 91% SUCRA values, respectively. For inflammatory lesions reduction, nineteen interventions were significantly better than placebo, and three interventions (TR+BPO: MD = −12.13, 95%CrI [−18.41, −5.80]; TR+BPO+MTCAM: −13.21 [−.39, −3.04]; LED: −11.30 [−18.34, −4.42]) were superior to others (SUCRA: 81, 81, and 77%, respectively). In summary of noninflammatory and inflammatory lesions results, TR+BPO and TA+BPO were the best options compared to others. The frequentist model showed similar results as above. In summary, current evidence supports the suggestion that TR+BPO and TA+BPO are the best options for mild to moderate acne. LED is another option for inflammatory lesions when drug resistance occurs. All the combinations involved with OA showed more risk of adverse events than others. However, the evidence of this study should be cautiously used due to the limitations.

## Introduction

Acne is a chronic inflammatory disease of pilosebaceous units resulting from androgen-induced increased sebum production, altered keratinization, inflammation, and bacterial colonization of hair follicles on the face, neck, chest, and back by *Cutibacterium acnes* (Williams et al., 2012). Nearly 80% of Americans will suffer from acne at some point in life time (Liu et al., 2015), and more than 50 million are facing this issue in the USA (White, 1998). In China, acne population prevalence is around 40%, and the rate rises to 50% in adolescents (Li et al., 2017). According to the study of the Global Burden of Disease, the acne population worldwide is 650 million, which made it one of the top 10 globally most prevalent diseases in 2010 (Hay et al., 2014). Acne affects adolescents worldwide (Rademaker et al., 1989; Yahya, 2009; Law et al., 2010; Wolkenstein et al., 2018). Although acne usually occurs in adolescence, more than half of patients will carry it into adulthood despite treatment (Bhate and Williams, 2013). However, studies on the natural course of acne are insufficient, and high-quality cohort studies are needed to provide evidence for that (Williams et al., 2012). On account of the insufficient evidence of the natural course of acne, potential factors that may affect the persistence of acne are not clear yet (Thiboutot et al., 2009).

General effects of acne are body feeling symptoms such as cutaneous pruritus and pains. However, the most severe impact is on the quality of life (Marron et al., 2019). This impact was influenced by various factors which made it hard to be quantitatively analyzed (Ayer and Burrows, 2006). The incidence rate reaches the highest in adolescents, a susceptible period of confidence and self-esteem, which may bring a persistent negative impact on adulthood. Studies reported that acne causes psychological and social abnormalities, such as depression, suicidal ideation, anxiety, psychosomatic symptoms, shame, embarrassment, and social inhibitions (Kubota et al., 2010; Bhate and Williams, 2013; Gieler et al., 2015). Moreover, these can be improved with treatment (Huang and Cheng, 2017; Li et al., 2019).

Along with the prevalence rate, disease burden and economic burden increase annually (GBD 2016 Disease and Injury Incidence and Prevalence; Collaborators, 2017; Karimkhani et al., 2017). In the United States, the annual direct cost of acne rose from one billion dollars in 2001 to 2.5 billion dollars in 2004. Among these, more than 100 million dollars were spent on over-the-counter acne products (James, 2005; Bickers et al., 2006; Bhate and Williams, 2013).

Conventional therapy includes topical treatments, oral treatments, complementary and alternative medications (CAM), miscellaneous therapies, and physical modalities. Although most therapies recommended by guidelines were supported by evidence from high-quality randomized control trials (Asai et al., 2016; Zaenglein et al., 2016), there still exist many areas of controversy and uncertainty (Williams et al., 2012). Debates mainly concentrated on the safety issue of retinoids, insufficient evidence for physical modalities and CAM, and antibiotic resistance. With the growth of antibiotic resistance, effective nonantibiotic therapies are badly needed. Despite this, the evidence for clinical efficacy of other than retinoids and benzoyl peroxide is not enough. Some studies reported good efficacy of lasers and photodynamic therapy, and so does chemical peels and some CAMs. However, none of these studies provided comparative efficacy, which resulted in an unavailable clinical decision making to share (Williams et al., 2012).

Therefore, we reevaluated all the available therapies through a network meta-analysis (Rücker, 2012; van Valkenhoef et al., 2012), which included pharmacological and nonpharmacological treatments, and compared the efficacy and safety of different therapies for the management of acne.

## Materials and Methods

We followed the Preferred Reporting Items for Systematic Reviews and Meta-Analyses (PRISMA) and the extension statement for network meta-analysis (Moher et al., 2009; Hutton et al., 2015). This systematic review and NMA protocol were registered with PROSPERO (CRD42019122413). Furthermore, we did some amendments relating to primary endpoints, statistical methodology, and including and excluding criteria during the research process, whose details were listed in Supplementary Material, File S1.

### Eligibility Criteria

Only randomized controlled trials (RCTs) for acne vulgaris were enclosed. Interventions of interest were as follows: pharmaceutical therapy: topical antibiotics (clindamycin, erythromycin, dapsone, etc.), benzoyl peroxide, topical retinoids, oral antibiotics (tetracycline, doxycycline, minocycline, etc.); physical modalities: lasers and light devices, chemical peels; miscellaneous therapies or complementary and alternative medicine; and the combinations of these mentioned above. Hormonal agents, such as combined oral contraceptives, flutamide, and oral corticosteroids, were excluded.

Except for children (younger than 12 years old), people of all ages with a diagnosis of acne vulgaris using clinical diagnosis or validated diagnostic criteria were included. Also, <4 weeks of follow-up and two weeks of treatment duration, with the diagnosis of “acne rosacea” and crossover trials and those that were published before 1985, were excluded. All included studies must clearly report at least one endpoint that we are concerned with, that is, amounts of inflammatory and noninflammatory lesions or numbers of subjects with adverse events.

### Literature Search

We searched PubMed, Embase, and the Cochrane Central Register of Controlled Trials (CENTRAL) from inception till April 2019 with “acne vulgaris” and “randomized controlled trials”, using a combination of Medical Subject Headings term and free-text terms. A complete search strategy is appended in Supplementary File S2.

### Study Process

According to eligibility criteria, two researchers (QS, LT) finished literature screening individually with respect to titles, abstracts, and full texts and evaluated the risk of bias for each article following the Cochrane handbook. CL assessed disagreements.

### Risk of Bias Assessment

Two researchers (QS, LT) independently appraised the risk of bias for included studies following the Cochrane risk of bias tool (Higgins and Green, 2011). The following aspects were assessed: random sequence generation, allocation concealment, blinding of participants and personnel, blinding of outcome assessment, incomplete outcome data, selective reporting, lost to follow-up number, intention-to-treat (ITT) analysis or not, multicenter trial or not, having a protocol or not, conflict of interests.

### Data Extraction

We extracted information of included RCTs from the following considerations: study characteristics (sample size of the trial, whether the trial was a multicenter study, length of follow-up, whether the trial had a protocol before it started, and whether the trial was a split-face study); patient characteristics (age, the proportion of women, and course of the disease); intervention characteristics (the type of retinoids and antibiotics, duration and dose of treatment); outcome data (count of noninflammatory and inflammatory lesions, numbers of subjects with adverse events).

### Outcomes

Primary outcomes were the mean change in noninflammatory and inflammatory lesions count from baseline. The lesions counts were extracted from the trials from tables or results when available or calculated from graphs using GetData Graph Digitizer (v2.26). The secondary outcome was the response rate of adverse events.

### Groups of Interventions

All the interventions were grouped into 30 categories: 15 single interventions and 15 combinations of interventions. The 15 single interventions and their abbreviations were classified as follows:

Topical therapies: topical antibiotics (TA) (included clindamycin, erythromycin, topical minocycline, and topical nadifloxacin); topical retinoids (TR) (adapalene, tretinoin, topical isotretinoin, and tazarotene); benzoyl peroxide (BPO); chemical peels (CP) (salicylic acid, azelaic acid, glycolic acid, and lipophilic hydroxy acid); topical dapsone (TD); topical tyrothricin (TT); topical spironolactone (TS); topical nicotinamide (TN).

Systemic antibiotics: oral antibiotics (OA) (minocycline, tetracycline, doxycycline, lymecycline, and faropenem).

Physical modalities: lasers (pulsed-dye laser, nonablative fractional laser, and fractionated erbium glass laser); LED devices (LED) (red and blue LED lights); photodynamic therapy (PDT); intense pulsed light (IPL).

Miscellaneous therapies or complementary and alternative medicine (MTCAM): Eladi Keram (Ayurvedic medicine); Olumacostat glasaretil (a novel topical sebum inhibitor); seaweed-derived oligosaccharide and zinc; triethyl citrate and ethyl linoleate; omega-3 fatty acid; gamma-linolenic acid; a decaffeinated green tea extract; sunflower seeds; chloroxylenol and zinc oxide.

### Statistical Analysis

A Bayesian method was used to perform pairwise meta-analyses and network meta-analyses. To account for the between-study heterogeneity and to attain greater generalizability for pooled results, all the analyses were carried out under a random effect model. The network model was performed under consistency assumption, and a node-splitting analysis was used to examine this assumption with presented pooled direct and indirect estimates and inconsistency *p* values for each split comparison (van Valkenhoef et al., 2016). The primary outcomes, namely, the mean change in noninflammatory and inflammatory lesions count from baseline, were calculated as mean difference (MD), and the secondary outcome, response rate of adverse events, was calculated as (log) risk ratio (RR).

We optimized the model and generated posterior samples using Markov Chain Monte Carlo methods running in four chains. We set at least 20,000 adaptation iterations to get convergence and 100,000 simulation iterations with a thinning factor of 10 to produce the outputs. We used the Brooks-Gelman-Rubin method to assess convergence of the model and calculated the “potential scale reduction factor” for each comparison together with the confidence interval (Gelman and Rubin, 1992; Brooks and Gelman, 1998). Approximate convergence is diagnosed when the upper limit was close to 1.

We presented the network estimates (pooled of direct and indirect data) of each intervention compared with placebo and each other in forest plot and league table. The median MD and RR of posterior estimates were reported with their 95% credible intervals (95%CrI). We also ranked interventions by their posterior probability by calculating the Surface Under the Cumulative Ranking (SUCRA) curve values (Salanti et al., 2011). And we presented their median ranks along with the 95%CrIs. To show the simultaneous assessment of heterogeneity, we conducted an analysis of heterogeneity for the network with both direct and indirect results and quantitated it as I-square, which was calculated from Cochran’s Q value (Higgins et al., 2003).

To lower the impact of potential effect modifiers in the network from a continuous covariate or a subgroup effect or the baseline risk, we conducted the Bayesian metaregression analysis to examine the robustness of effect estimates of primary outcomes with nine covariates (Dias et al., 2013). We additionally conducted a sensitivity analysis with an alternative vague prior (*σ* ∼ Unif (0, 5)) or a weakly information prior (*τ* ∼ HN (1)) or an empirical prior for a subjective outcome with any intervention (*τ* ∼ Lognormal (-2.01, 0.372)) as recommended by Rhodes et al. (2015).

We also performed a network meta-analysis using frequentist methods with random effects based on the graph-theoretical method (Rücker, 2012). Afterward, we conducted an additive component network meta-analysis for combinations of treatments (Rücker et al., 2019). The rank of treatments was calculated by *P*-scores (Rücker and Schwarzer, 2015). To find potential publication bias and small-study effects, we performed the comparison-adjusted funnel plots with the specified order by *P*-scores (Chaimani and Salanti, 2012). In addition, we performed a pairwise meta-analysis using a random effect method for comparison of miscellaneous therapies or complementary and alternative medicine vs. placebo.

All computations were done using R (V. 3.5.2) package gemtc (Valkenhoef, 2018) (V. 0.8–2) along with the Markov Chain Monte Carlo engine JAGS (V. 3.4.0) and package netmeta (Schwarzer, 2019) (V. 1.0–1) and package metafor (Viechtbauer, 2019) (V. 2.0–0). We performed the risk of bias graph using the Cochrane tool RevMan (V. 5.3). The complete statistical analysis is in Supplementary File S3.

## Result

### Literature Review

**FIGURE 1:**
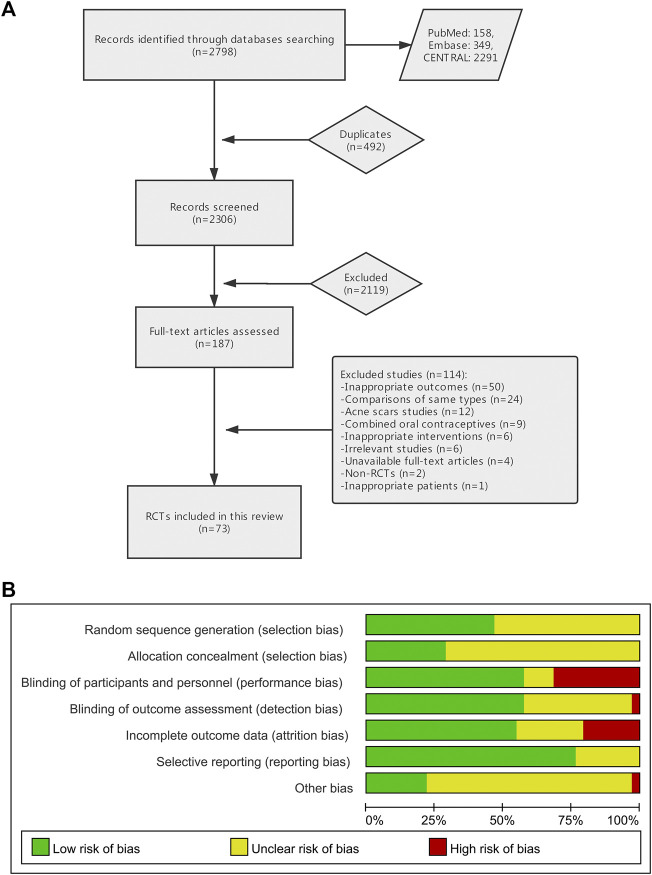
**(A)** Flow diagram of selection of studies. **(B)** Risk of bias graph.

Of 2,798 references identified, 187 were initially included for full-text assessed. Of these, 114 studies were excluded for nine reasons, and 73 studies were finally included in quantitative synthesis (Figure 1A) (Supplementary Table S1). In total, 27,745 patients were enrolled with mild to moderate acne, and the mean age was 21.5 with a range of 12–50. Studies were conducted in 29 countries, of which 38 were multicenter trials. The mean duration of treatment was 10.5 weeks with a range of 2–24.

We presented the risk of bias graph and table in Figure 1B and Supplementary Table S2. Thirty-four trials (46.6%) reported the method of random sequence generation, and 21 (28.8%) reported concealed treatment allocation. Forty-two trials (57.5%) informed blinded participants and personnel, and 42 (57.5%) informed blinded investigators. Forty trials (54.8%) had low risk in attrition bias, and 56 (76.7%) had that in reporting bias.

### Comparative Effects on Noninflammatory Lesions Count

**FIGURE 2:**
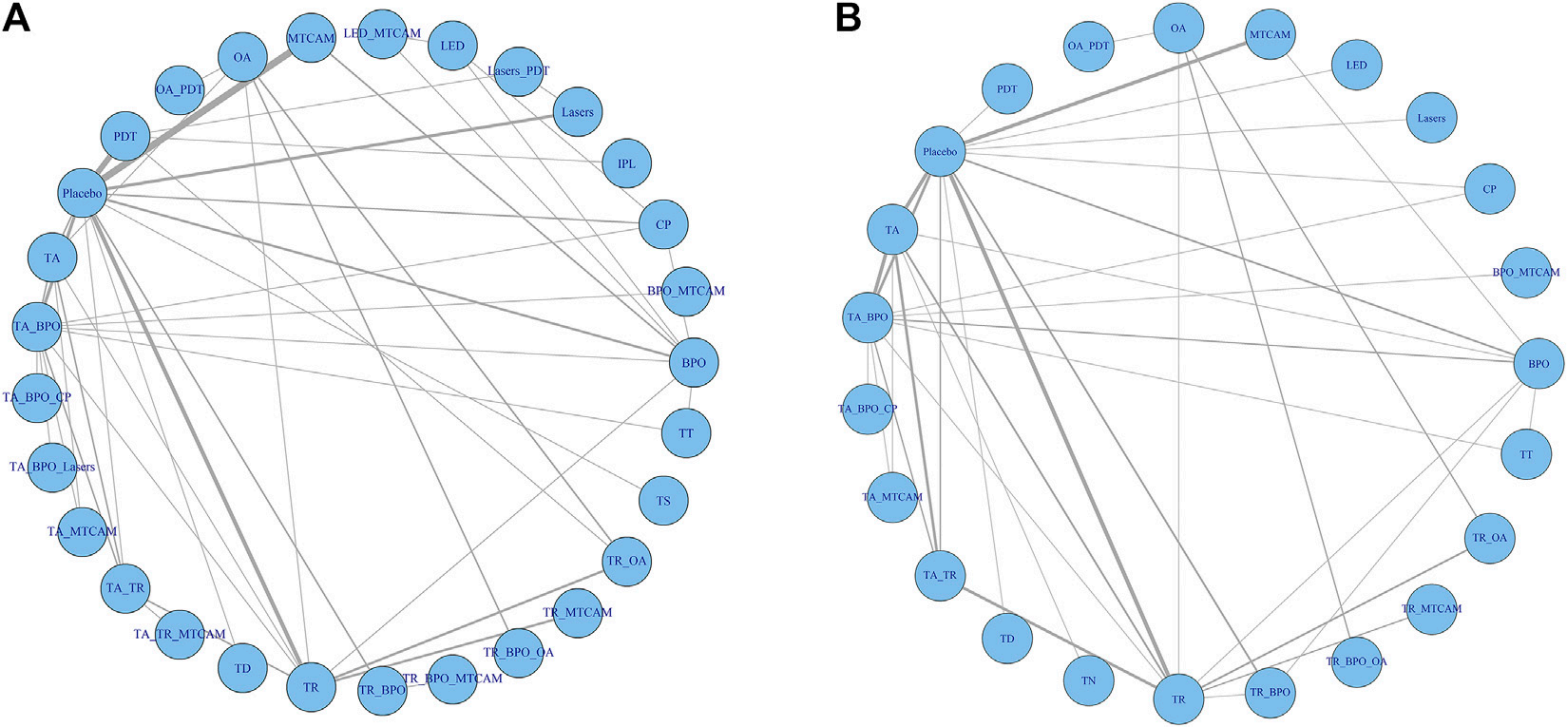
Network analysis plots. **(A)** Noninflammatory and inflammatory lesions count. **(B)** Risk of adverse effects.

**FIGURE 3:**
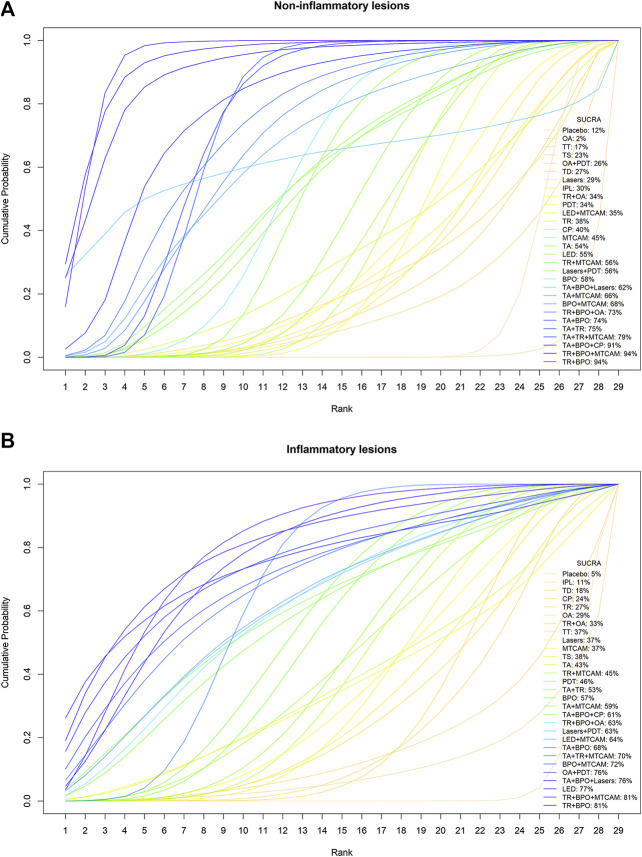
Cumulative probability curves and SUCRA values. **(A)** Noninflammatory lesions count. **(B)** Inflammatory lesions count.

**FIGURE 4:**
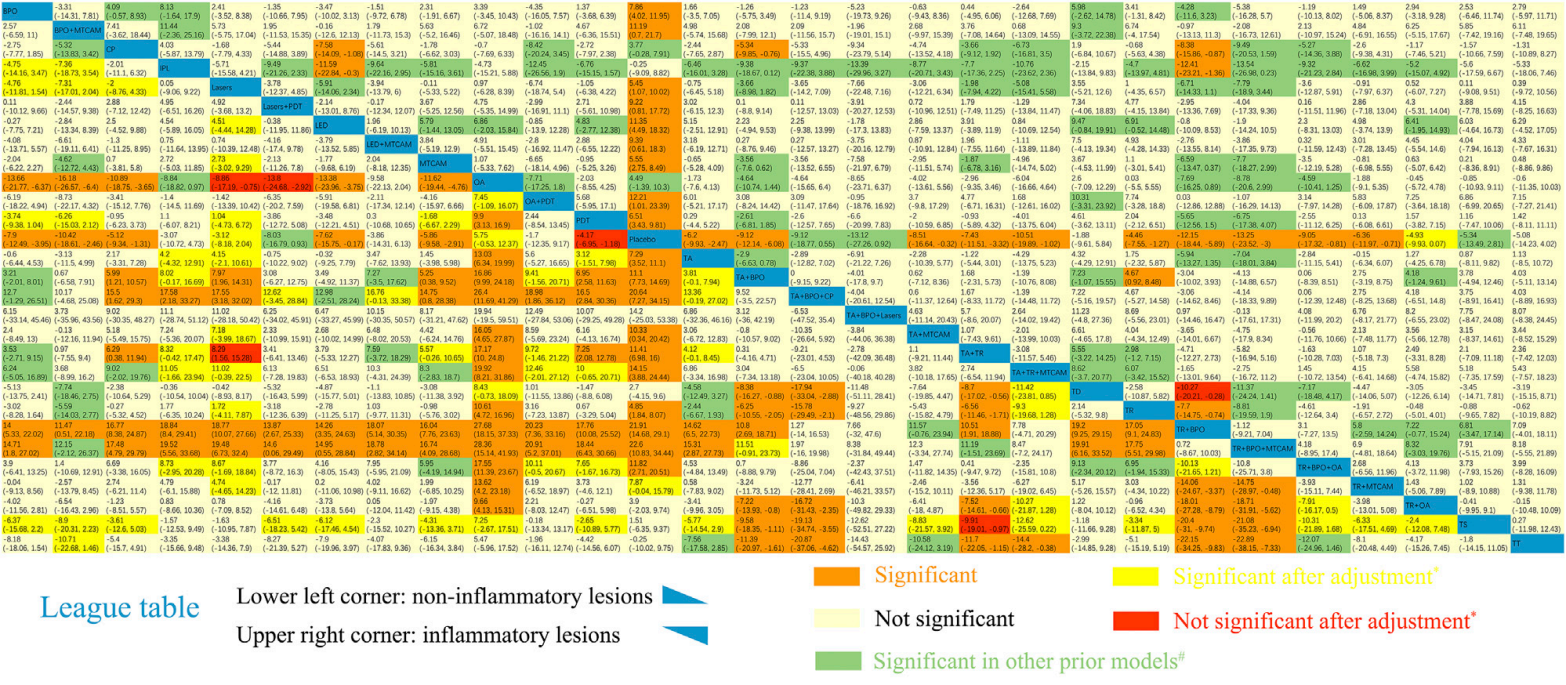
League table of NMA estimations. Lower-left corner: Mean difference of noninflammatory lesions count. Upper-right corner: Mean difference of inflammatory lesions count. *The results became significant after adjustment via metaregression. #The results became significant in other prior models. Comparisons should be read from up to right in the lower-left corner or from down to left in the upper-right corner.

Sixty-one trials were included in the network of noninflammatory lesions count, involving 149 treatment arms and 17,306 patients. The net graph of treatment comparisons of these studies was shown in Figure 2A. Fourteen interventions had a significant reduction than placebo (TR+BPO: MD = −21.89, 95%CrI [−28.97, −14.76]; TR+BPO+MTCAM: −22.48 [−34.13, −10.70]; TA+BPO+CP: −20.63 [−33.97, −7.13]; TA+TR+MTCAM: −14.15 [−24.43, −3.97]; TA+TR: −11.42 [−15.91, −7.02]; TA+BPO: −11.10 [−14.61, −7.75]; TR+BPO+OA: −11.84 [−20.52, −2.98]; BPO+MTCAM: −10.44 [−18.52, −2.59]; BPO: −7.84 [−12.42, −3.95]; TA: −7.29 [−10.99, −3.55]; MTCAM: −5.82 [−9.62, −2.96]; CP: −5.08 [−9.26, −1.33]; TR: −4.86 [−7.99, −1.88]; PDT: −4.19 [−6.92, −1.19]) and another three interventions had a borderline significance (TA+MTCAM: −10.17 [−20.13, −0.31]; TR+MTCAM: −7.92 [−15.81, −0.04]; LED: −7.50 [−15.54, −0.17]) (S2 Page 1). In the SUCRA graph (Figure 3A), three interventions were obviously superior to others, namely, TR+BPO, TR+BPO+MTCAM, and TA+BPO+CP (SUCRA: 94, 94, and 91%, respectively). All interventions compared with each other were shown in league table in Figure 4 lower-left corner.

In the node-splitting analysis, only a comparison between BPO and placebo showed a significant difference of inconsistency between direct and indirect comparisons (*p* value = 0.0006) (S2 Page 2). In the model fitting graph of leverage vs. residual deviance, we found one fitting observation (Charakida et al., 2007), representing the comparison of MTCAM vs. placebo, which was far from the others. In the analysis of heterogeneity (S2 Page 3), we detected high heterogeneity of pairwise pooled effect (more than three trials pooled) in four comparisons, involving placebo vs. MTCAM, placebo vs. PDT, TA+BPO vs. placebo, and TR vs. placebo (I-square: 96, 70.4, 72.2, and 84.1%, respectively).

### Metaregression and Sensitivity Analysis

Only two covariates showed a significant coefficient in interaction model (duration of treatment: *β* = −3.9955, 95%CrI [−6.792, −0.5273]; whether it is of low quality or not: −10.668 [−19.231, −2.2532]) (Supplementary Table S3). That means MD decreases along with the longer duration of treatment or within low-quality studies. In the model of adjusted NMA with the covariates centering, we found some interventions partially changed their 95%CrI (Figure 4 lower-left corner). And five interventions changed their SUCRA values, involving lasers+PDT, PDT, IPL, lasers, and TS (Supplementary Table S4).

In the sensitivity analysis with different priors (narrower uniform, lognormal and half-normal distribution), we found all included interventions had a narrower credible interval compared with the original model, which means some of them changed their 95%CrI in a significant reduction (Figure 4 lower-left corner). The half-normal prior model had the narrowest credible interval, followed by lognormal and uniform prior. Three interventions changed their SUCRA values in the half-normal prior model, including BPO, MTCAM, and LED+MTCAM (Supplementary Table S4).

### Frequentist Additive Network Meta-Analysis

**FIGURE 5:**
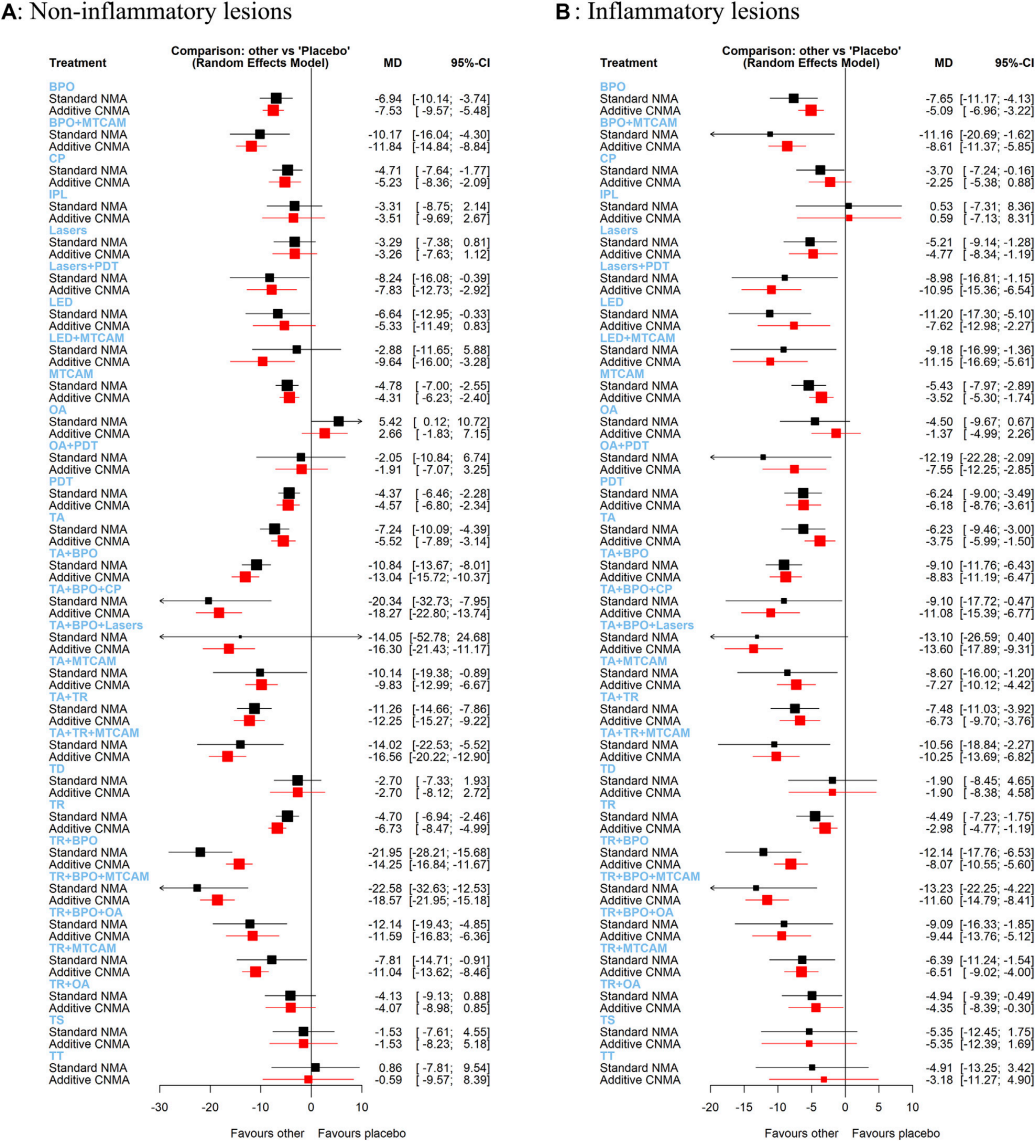
Forest plots of frequentist additive network meta-analysis. **(A)** Mean difference of noninflammatory lesions count. **(B)** Mean difference of inflammatory lesions count.

In both standard NMA and additive NMA, results were similarly shown as that in the Bayesian model. However, two interventions (TA+BPO+lasers and LED+MTCAM) changed their 95%CI in a significant reduction compared with placebo in the additive NMA model (Figure 5A). And because of increased available treatment arms and the probable interaction effects in the combination of two or more treatments, some interventions had a narrower or changed confidence interval at the same level. Two interventions (TR+BPO and TR+BPO+MTCAM) had significant reductions of MD in standard NMA than that in additive NMA. Conversely, two interventions (LED+MTCAM and TR+MTCAM) had fewer reductions in standard NMA than the other.

### Effects of MTCAM Versus Placebo

**FIGURE 6:**
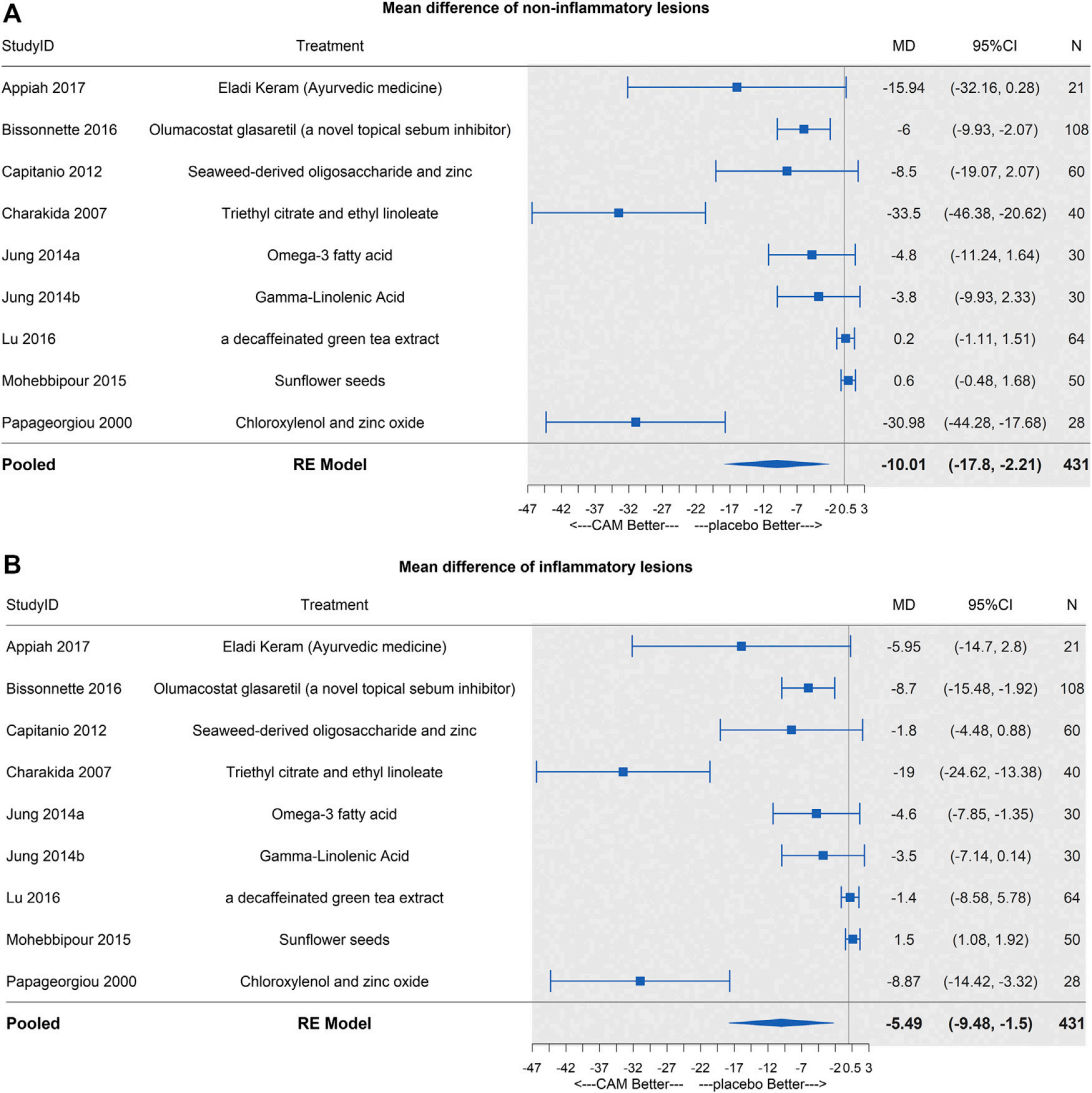
Forest plots of MTCAM vs. placebo. **(A)** Mean difference of noninflammatory lesions count. **(B)** Mean difference of inflammatory lesions count.

In the pairwise meta-analysis, only three trials were statistically significant at the 0.05 level (Figure 6A). So, we conducted a leave-one-out analysis and detected a borderline significance on mean difference when excluding Charakida et al. (2007) or Papageorgiou and Chu (2000) (95%CI [−12.54, −0.82]; [−13.98, −0.69], respectively) (S2 Page 4A).

### Comparative Effects on Inflammatory Lesions Count

Sixty-two trials were included in the network of inflammatory lesions (151 arms and 17,490 patients) and the net graph was shown in Figure 2A (same as noninflammatory lesions). Twelve interventions had a significant reduction (TR+BPO: MD = −12.13, 95%CrI [−18.41, −5.80]; TR+BPO+MTCAM: −13.21 [−23.39, −3.04]; LED: −11.30 [−18.34, −4.42]; TA+TR+MTCAM: −10.48 [−19.76, −1.02]; TA+BPO: −9.08 [−12.14, −6.02]; BPO: −7.85 [−11.97, −3.96]; TA+TR: −7.39 [−11.52, −3.27]; PDT: −6.48 [−9.79, −3.45]; TA: −6.17 [−9.91, −2.45]; MTCAM: −5.53 [−8.46, −2.67]; lasers: −5.44 [−9.94, −1.10]; TR: −4.43 [−7.54, −1.25]) and the other seven interventions had a borderline significance (OA+PDT: −12.13 [−23.20, −0.83]; BPO+MTCAM: −11.09 [−21.52, −0.69]; LED+MTCAM: −9.30 [−18.18, −0.64]; lasers+PDT: −9.15 [−17.63, −0.78]; TR+BPO+OA: −9.00 [−17.22, −0.76]; TA+MTCAM: −8.53 [−16.59, −0.41]; TR+MTCAM: −6.33 [−11.89, −0.74]) (S2 Page 5). In the SUCRA graph (Figure 3B), two interventions (TR+BPO and TR+BPO+MTCAM) were superior to others, followed by LED, TA+BPO+lasers, and OA+PDT (SUCRA: 81, 81, 77, 76, and 76%, respectively). All interventions compared with each other were shown in league table in Figure 4 upper-right corner.

In the node-splitting analysis (S2 Page 6), three comparisons had a significant difference between direct and indirect results (TR+OA vs. PDT; TR vs. placebo; TR+OA vs. TR, *p* value = 0.03; 0.035; 0.048, respectively). And we did not identify the influential or poorly fitting observations in the leverage and residual deviance graph. However, we detected the significant heterogeneity of pairwise pooled effect in four comparisons, including placebo vs. lasers, placebo vs. MTCAM, placebo vs. PDT, and TR vs. placebo (I-square: 97.2, 99.2, 93.2, and 77.7%, respectively) (S2 Page 7).

### Metaregression and Sensitivity Analysis

In the interaction model, we detected two covariates with significant coefficients (whether it is double-blinded or not: *β* = −9.653, 95%CrI [−13.2, −6.28]; whether it is a split-face study or not: 5.79 [0.84, 11.1]), which means effect size may be overestimated in non-double-blinded studies or split-face studies (Supplementary Table S5). In the adjusted NMA model, only two comparisons significantly changed their 95%CrI (Figure 4 upper-right corner), and no intervention changed their SUCRA values (Supplementary Table S6).

In the sensitivity analysis, which is the same as noninflammatory lesions, narrower credible intervals were found, and some of them changed their 95%CrI (Figure 4 upper-right corner). Three interventions changed their SUCRA values in the half-normal prior model (PDT, lasers, and IPL) (Supplementary Table S6).

### Frequentist Additive Network Meta-Analysis

We found similar results as that of the Bayesian model in both standard NMA and additive NMA, but one intervention (TA+BPO+lasers) changed 95%CI in a significant reduction compared with placebo (Figure 5B). Three interventions (BPO+MTCAM, OA+PDT, and TR+BPO) had more significant reductions in standard NMA than that in additive NMA, and three interventions (lasers+PDT, LED+MTCAM, and TA+BPO+CP) had fewer reductions.

### Effects of MTCAM Versus Placebo

Similar results were shown as noninflammatory lesions, and three same trials were significant (Figure 6B). In the leave-one-out analysis, borderline significances were detected after (Bissonnette et al., 2017), (Charakida et al., 2007), or (Papageorgiou and Chu, 2000) (95%CI [−9.55, −0.79]; [−6.14, −0.72]; [−9.51, −0.72], respectively) were excluded (S2 Page 4B).

### Summary of the Relative Efficacy of Noninflammatory and Inflammatory Lesions

**FIGURE 7:**
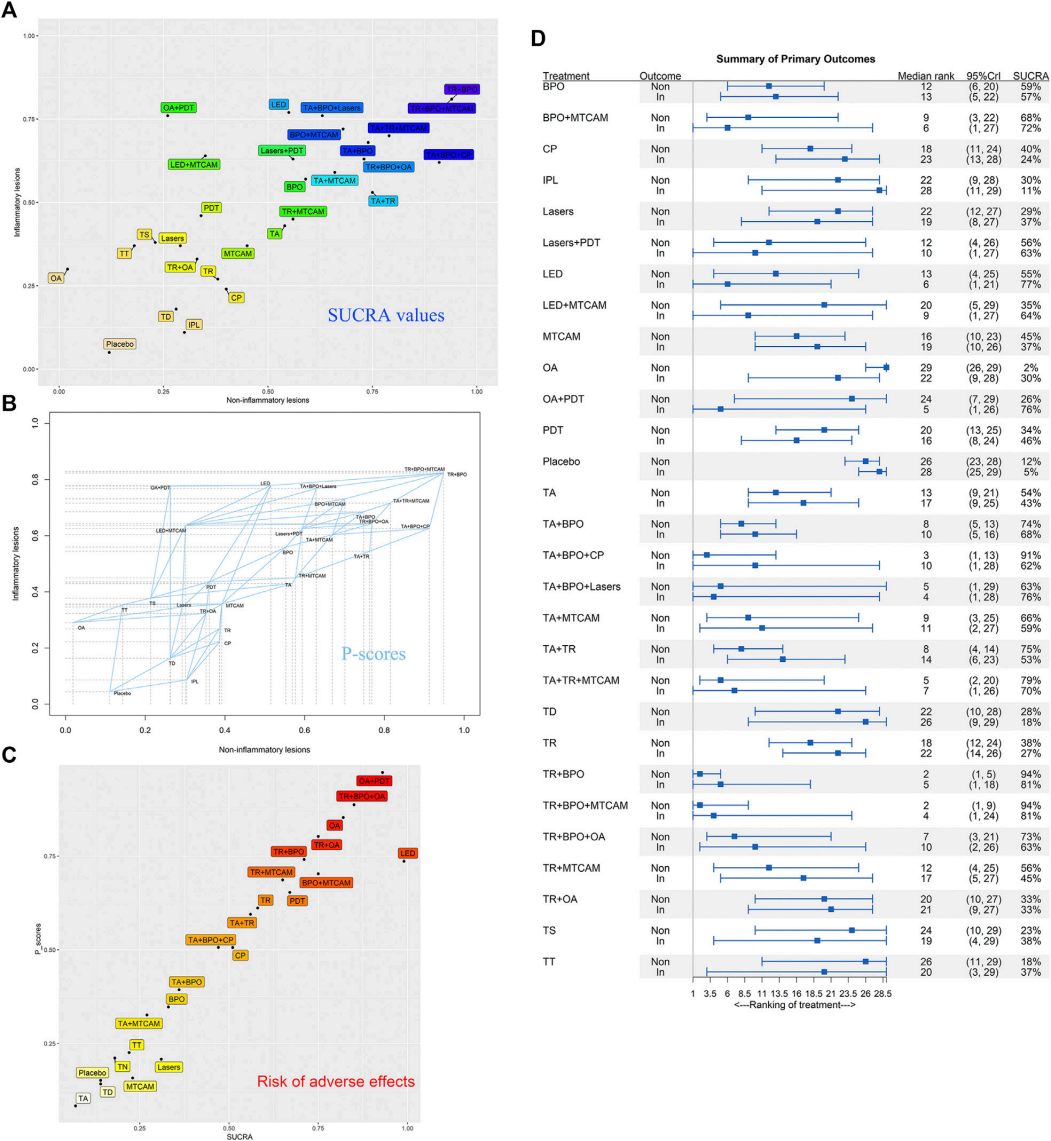
Biplots of SUCRA values and P-scores for efficacy and safety outcomes. **(A)** SUCRA values of noninflammatory and inflammatory lesions count. **(B)** P-scores of noninflammatory and inflammatory lesions count. **(C)** SUCRA values and P-scores of adverse effects. **(D)** Median rank and 95%CrI of summarized primary outcomes.

We synthesized the primary outcomes of noninflammatory and inflammatory lesions in biplots based on SUCRA values in Bayesian NMA and P-scores in frequentist NMA, respectively. And the similar results were shown in both models (Figures 7A,B). Besides, the median rank and corresponding 95%CrI were displayed in Figure 7D. TR+BPO showed the largest lesions reduction in both noninflammatory and inflammatory lesions. TR+BPO+MTCAM had similar efficacy, but with more components it may bring additional risk. In the funnel plot analysis, we detected that published bias might exist in both noninflammatory and inflammatory lesions result, but Egger’s regression test and rank correlation test showed inconsistent results (S2 Page 10).

### Comparative Risks of Adverse Effects

Fifty trials were included in the network of response rates with adverse events, involving 112 treatment arms and 25,437 patients. The net graph was shown in Figure 2B. In the Bayesian NMA, except for three interventions that had similar risk compared with placebo, all the others showed more adverse effects than placebo, and fourteen of them (BPO, BPO+MTCAM, CP, LED, OA, OA+PDT, PDT, TA+BPO, TA+TR, TR, TR+BPO, TR+BPO+OA, TR+MTCAM, and TR+OA) had a significant difference (S2 Page 8A). In the node-splitting analysis, only one comparison had a significant difference between direct and indirect results (TR vs. OA: *p* value = 0.034) (S2 Page 9). In the frequentist NMA, similar results were shown (S2 Page 8B). In both SUCRA and P-scores, it was shown that three interventions had more risk of adverse effects than others, which included OA+PDT, TR+BPO+OA, and OA (Figure 7C). In the funnel plot analysis, no published bias was found in all asymmetry tests (S2 Page 10C).

## Discussion

### Main Findings

In this network meta-analysis, we pooled evidence from 73 studies (27,745 patients with mild to moderate acne). Due to some missing data, 62 of them reported primary outcomes, and 44 studies reported secondary outcomes.

For noninflammatory lesions reduction, we found that seventeen interventions had a significant reduction than placebo. TR+BPO, TR+BPO+MTCAM, and TA+BPO+CP were superior to others with 94%, 94%, and 91% SUCRA values, respectively (TR+BPO: MD = −21.89, 95%CrI [−28.97, −14.76]; TR+BPO+MTCAM: −22.48 [−34.13, −10.70]; TA+BPO+CP: −20.63 [−33.97, −7.13]). In the frequentist NMA, besides the similar results as that of Bayesian NMA, two interventions (TR+BPO and TR+BPO+MTCAM) may have potential positive interaction effect due to a more significant reduction in standard NMA than that in additive NMA.

For inflammatory lesions reduction, we found that nineteen interventions were statistically significant. TR+BPO, TR+BPO+MTCAM, and LED were superior to others with 81, 81, and 77% SUCRA values, respectively (TR+BPO: MD = −12.13, 95%CrI [−18.41, −5.80]; TR+BPO+MTCAM: −13.21 [−23.39, −3.04]; LED: −11.30 [−18.34, −4.42]). Besides similar results as that of the Bayesian model, three interventions (BPO+MTCAM, OA+PDT, and TR+BPO) may have potential positive interaction effects.

Although MTCAM showed a significant reduction in both noninflammatory and inflammatory lesions, its result was unrobust (a borderline significance) in leave-one-out analysis in the pairwise analysis because of different effect sizes in different types of treatment.

Based on the summary of evidence for noninflammatory and inflammatory lesions, we considered that TR+BPO was the best option compared to the others. TA+BPO and TA+TR were also good options compared with others. These three interventions are consistent with guideline recommendations for mild and moderate acne. However, the last two interventions showed that they had significant adverse effects compared with placebo. Thus, TR+BPO may be the preferred option due to lack of antibiotics. When drug resistance occurs, LED seemed to be the better option either alone or in adjuvant treatment. But there is still a need for further studies to support this suggestion. For adverse effects, OA had more risk in combination treatment with others, and the efficacy was not so good. Thus, we considered that OA should not be used hastily and require a trade-off between efficacy and risk of adverse effects in clinical practice. According to current evidence, MTCAM should not be considered as a treatment strategy until more high-quality studies are available to demonstrate its efficacy.

### Strengths and Limitations

It is the first network meta-analysis of different interventions for acne, which adopted multiple data synthesis approaches, including Bayesian and frequentist models. Various statistical methods allow us to accomplish a comprehensive study on the data under different suitable conditions, from which more convincing results were provided.

### 

In the Bayesian model, firstly, in the lack of included study amounts, we conducted a noninformative prior model to obtain a wide credible interval for results. To estimate the potential statistically significant results from the above ones, we compared the differences between results generated by applying different informative prior assumptions in the sensitivity analysis. The result from half-normal distribution provides the narrowest credible interval, which made it the upper bound estimate while the result from the original model was the lower ones.

Secondly, we ranked all interventions according to their SUCRA values and analyzed primary outcomes with a biplot graph to provide clinical suggestions. Then, node-splitting analysis and heterogeneity analysis were used to evaluate the inconsistency between direct and indirect comparison and heterogeneity in direct comparison.

Finally, we conducted the metaregression against the potential impact of effect modifiers and adjusted the covariates with significant coefficients. Some variations occurred then that the differences between some interventions in noninflammatory lesions became statistically significant, and some SUCRA values changed. However, these variations showed little impact on the robustness of results because the ranking of top-five interventions remained unchanged.

In the frequentist model, the outcomes of standard network meta-analysis were similar to that of the Bayesian model, which indicated a robust final result. We also did additive network meta-analysis to evaluate the potential interaction effects of compounds. Combined interventions such as TR+BPO, TR+BPO+MTCAM, BPO+MTCAM, and OA+PDT presented positive interaction effects, which indicated more benefits in efficacy from these combinations. Basing on P-score and SUCRA values, respectively, the two biplots synthesized of two primary outcomes confirmed the consistency of final efficacies, which indicated a similar result from two different methods.

There exists a significant heterogeneity in this study, for we combined several MTCAM when doing network analysis. To detect the influence, we conducted a pairwise meta-analysis and leave-one-out analysis. Two treatments (triethyl citrate + ethyl linoleate and chloroxylenol + zinc oxide) are the resources of heterogeneity for their efficacy being much superior to others. The overall efficacy of MTCAM is unrobust, which requires further study to evaluate its combined efficacy.

Quality of included studies varied from moderate to high, and only seven studies (Katsambas et al., 1987; Bojar et al., 1994, 199; Papageorgiou and Chu, 2000; Afzali et al., 2012; Moneib et al., 2014; Alba et al., 2017, 201; Lekwuttikarn et al., 2017) (10%) were judged as low quality with only one low risk of bias. High-risk judgments were mainly about “Blinding of participants and personnel” and “Incomplete outcome data”. Adjustments we conducted in the metaregression were against the impact of these biases.

There now exist no researches concerning about comparison between pharmacotherapy and nonpharmacotherapy, which is why we included these two classes of interventions together in this study. As the results showed, one nonpharmacotherapy (LED) was discovered with good efficacy in treating inflammatory lesions, which seemed to be nearly at the same level as first-line medications and superior to other medications. That may invoke more interests in further studies on the potential clinical efficacy of physical modalities. However, due to some limitations of the evidence, it should not be easily recommended.

Several limitations are present in this study. Firstly, out of all 73 included studies, 11 studies did not report inflammatory and noninflammatory outcomes, while 29 did not report the numbers of subjects with adverse events. Also, there may exist selection bias of included trials in this study because we excluded some studies for some certain reason: studies that reported no specific amounts of noninflammatory and inflammatory outcomes; studies concentrating on comparison of same kind interventions; studies that reported no standard deviation of primary outcomes.

Secondly, the results of three comparisons presented inconsistency in the node-splitting analysis of inflammatory lesions. However, the inconsistency *p* values were borderline significant, which may impact little on pooled results. Considering noninflammatory lesions, inconsistency in BPO vs. placebo is much higher. In other words, the efficacy is much higher in direct comparison than that in indirect one. Further studies were necessary to illustrate this inconsistency. Five comparisons showed higher heterogeneity values on inflammatory and noninflammatory lesions. The reason for comparisons between TR vs. placebo and placebo vs. PDT is that there existed a dose-response relationship, and we only used their average efficacies. The reason for the placebo vs. MTCAM is caused by including different interventions.

Thirdly, oral contraceptive was excluded to ensure the consistency of included studies, and oral isotretinoin was also excluded because of eligibility criteria. However, these are recommended in treating severe acne. Thus, a study focusing on that is necessary afterward.

Finally, practitioners who cite the conclusions of this study should pay attention to its extrapolation, for we considered different specific interventions as one group, such as topical antibiotics, lasers, chemical peels, photosensitizers with PDT, retinoids, and MTCAM, according to their classifications in our analysis. That is, the conclusions were more reliable when talking about these specific interventions we included rather than that we did not.

### Comparison With Other Studies

There were two meta-analyses studied on BPO, BPO+SA, BPO+CL, CL, and placebo (Seidler and Kimball, 2010; Seidler and Kimball, 2011). One research concluded that, in dealing with noninflammatory lesions, BPO+SA and BPO+CL are with similar efficacy, which is better than applying BPO or CL alone, while in inflammatory lesions, BPO+CL and CL showed similar efficacy, which is a little superior to that of BPO+SA or BPO. Another study believed BPO+CL has the best efficacy in both inflammatory and noninflammatory lesions, followed by BPO or CL alone. These are completely consistent with our conclusions.

A meta-analysis study in 2015 compared the efficacy of different types of CAM, suggesting that some CAMs may be effective, and most CAMs cannot be accurately judged because of the low quality of the primary literature (Cao et al., 2015). Unlike that study, we only included primary literature with higher quality and clearly defined inflammatory and noninflammatory lesions as outcome indicators. However, the results are alike, that is, CAM vs. placebo is borderline significant, which indicates an unclear conclusion of them being clinically effective or invalid.

Another meta-analysis in 2018 compared the efficacy of different types of chemical peels, concluding that the effects of different types of chemical peels are similar, maybe superior to placebo in noninflammatory lesions reduction, and there is insufficient evidence in the inflammatory lesions (Chen et al., 2018). That is also consistent with our conclusions.

Guideline of the American Academy of Dermatology in 2016 suggested that laser and light devices are possibly beneficial in treating acne but with insufficient evidence (Zaenglein et al., 2016). In our study, LED seemed to be comparable in efficacy to first-line medications in the treatment of inflammatory lesions, which is worth doing further studies focusing on light devices.

## Conclusions

For mild to moderate acne, we found TR+BPO appeared to be the best option, followed by TA+BPO. TA+BPO+CP and TA+TR were another two good options for noninflammatory lesions and LED was good for inflammatory lesions. For adverse effects, all the combinations involved with OA showed more risks than others. Due to the limitations, the evidence of this study should be cautiously used.

## Data Availability Statement

The original contributions presented in the study are included in the article/Supplementary Material; further inquiries can be directed to the corresponding authors.

## Author Contributions

Study design and conception were done by QS and CL. Literature searching and extraction of data were done by QS, ZC, LT, XZ, and FY. Analysis and interpretation of data were done by QS and LG. Drafting of the manuscript was done by QS and LT. Critical revision of the manuscript was done by QS, LT, XZ, CL, JZ, and LG. Study supervision and funding were done by CL and JZ.

## Funding

This study was supported by the National Natural Science Foundation of China (CN) (No. 81804218) and Training Program of Innovation Team of Tianjin Higher Education Institution through Tianjin Municipal Education Commission (No. TD13-5047).

## Conflict of Interest

The authors declare that the research was conducted in the absence of any commercial or financial relationships that could be construed as a potential conflict of interest.
