# Supplementary material for: Comparative Efficacy of Pharmacological and Nonpharmacological Interventions for Acne Vulgaris: A Network Meta-Analysis
Source: Front Pharmacol. 2020 Nov 26;11:592075. doi: 10.3389/fphar.2020.592075 (PMC7729523; doi:10.3389/fphar.2020.592075)

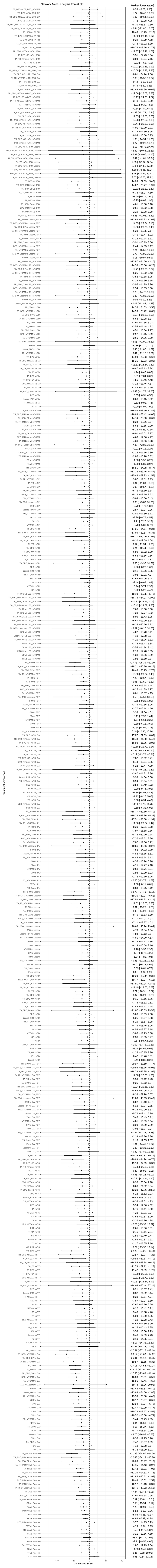
Network Meta-analysis Forest plot
Median [lower, upper]
Treatment comparison
Continuous Scale

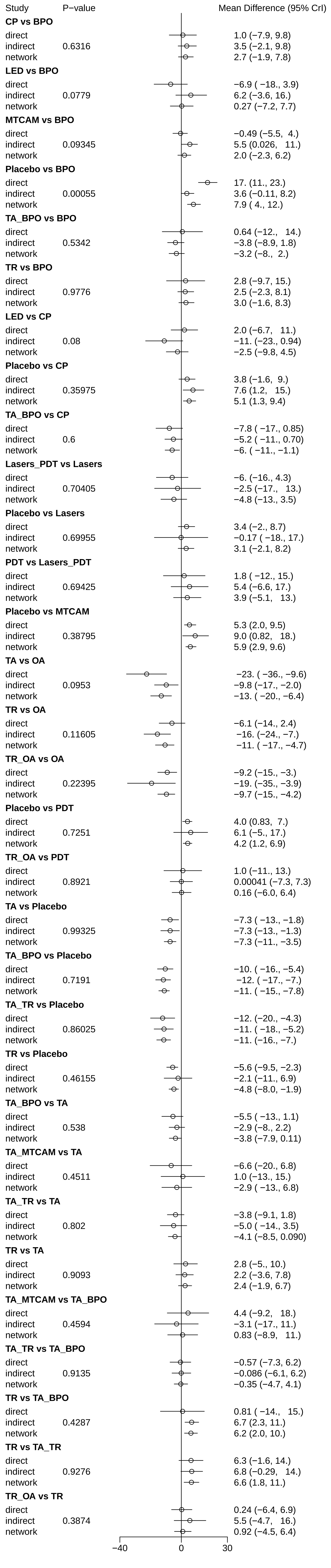
Study P−value Mean Difference (95% CrI)
CP vs BPO
direct 1.0 (−7.9, 9.8)
indirect 0.6316 3.5 (−2.1, 9.8)
network 2.7 (−1.9, 7.8)
LED vs BPO
direct −6.9 (−18., 3.9)
indirect 0.0779 6.2 (−3.6, 16.)
network 0.27 (−7.2, 7.7)
MTCAM vs BPO
direct −0.49 (−5.5, 4.)
indirect 0.09345 5.5 (0.026, 11.)
network 2.0 (−2.3, 6.2)
Placebo vs BPO
direct 17. (11., 23.)
indirect 0.00055 3.6 (−0.11, 8.2)
network 7.9 (4., 12.)
TA_BPO vs BPO
direct 0.64 (−12., 14.)
indirect 0.5342 −3.8 (−8.9, 1.8)
network −3.2 (−8., 2.)
TR vs BPO
direct 2.8 (−9.7, 15.)
indirect 0.9776 2.5 (−2.3, 8.1)
network 3.0 (−1.6, 8.3)
LED vs CP
direct 2.0 (−6.7, 11.)
indirect 0.08 −11. (−23., 0.94)
network −2.5 (−9.8, 4.5)
Placebo vs CP
direct 3.8 (−1.6, 9.)
indirect 0.35975 7.6 (1.2, 15.)
network 5.1 (1.3, 9.4)
TA_BPO vs CP
direct −7.8 (−17., 0.85)
indirect 0.6 −5.2 (−11., 0.70)
network −6. (−11., −1.1)
Lasers_PDT vs Lasers
direct −6. (−16., 4.3)
indirect 0.70405 −2.5 (−17., 13.)
network −4.8 (−13., 3.5)
Placebo vs Lasers
direct 3.4 (−2., 8.7)
indirect 0.69955 −0.17 (−18., 17.)
network 3.1 (−2.1, 8.2)
PDT vs Lasers_PDT
direct 1.8 (−12., 15.)
indirect 0.69425 5.4 (−6.6, 17.)
network 3.9 (−5.1, 13.)
Placebo vs MTCAM
direct 5.3 (2.0, 9.5)
indirect 0.38795 9.0 (0.82, 18.)
network 5.9 (2.9, 9.6)
TA vs OA
direct −23. (−36., −9.6)
indirect 0.0953 −9.8 (−17., −2.0)
network −13. (−20., −6.4)
TR vs OA
direct −6.1 (−14., 2.4)
indirect 0.11605 −16. (−24., −7.)
network −11. (−17., −4.7)
TR_OA vs OA
direct −9.2 (−15., −3.)
indirect 0.22395 −19. (−35., −3.9)
network −9.7 (−15., −4.2)
Placebo vs PDT
direct 4.0 (0.83, 7.)
indirect 0.7251 6.1 (−5., 17.)
network 4.2 (1.2, 6.9)
TR_OA vs PDT
direct 1.0 (−11., 13.)
indirect 0.8921 0.00041 (−7.3, 7.3)
network 0.16 (−6.0, 6.4)
TA vs Placebo
direct −7.3 (−13., −1.8)
indirect 0.99325 −7.3 (−13., −1.3)
network −7.3 (−11., −3.5)
TA_BPO vs Placebo
direct −10. (−16., −5.4)
indirect 0.7191 −12. (−17., −7.)
network −11. (−15., −7.8)
TA_TR vs Placebo
direct −12. (−20., −4.3)
indirect 0.86025 −11. (−18., −5.2)
network −11. (−16., −7.)
TR vs Placebo
direct −5.6 (−9.5, −2.3)
indirect 0.46155 −2.1 (−11., 6.9)
network −4.8 (−8.0, −1.9)
TA_BPO vs TA
direct −5.5 (−13., 1.1)
indirect 0.538 −2.9 (−8., 2.2)
network −3.8 (−7.9, 0.11)
TA_MTCAM vs TA
direct −6.6 (−20., 6.8)
indirect 0.4511 1.0 (−13., 15.)
network −2.9 (−13., 6.8)
TA_TR vs TA
direct −3.8 (−9.1, 1.8)
indirect 0.802 −5.0 (−14., 3.5)
network −4.1 (−8.5, 0.090)
TR vs TA
direct 2.8 (−5., 10.)
indirect 0.9093 2.2 (−3.6, 7.8)
network 2.4 (−1.9, 6.7)
TA_MTCAM vs TA_BPO
direct 4.4 (−9.2, 18.)
indirect 0.4594 −3.1 (−17., 11.)
network 0.83 (−8.9, 11.)
TA_TR vs TA_BPO
direct −0.57 (−7.3, 6.2)
indirect 0.9135 −0.086 (−6.1, 6.2)
network −0.35 (−4.7, 4.1)
TR vs TA_BPO
direct 0.81 (−14., 15.)
indirect 0.4287 6.7 (2.3, 11.)
network 6.2 (2.0, 10.)
TR vs TA_TR
direct 6.3 (−1.6, 14.)
indirect 0.9276 6.8 (−0.29, 14.)
network 6.6 (1.8, 11.)
TR_OA vs TR
direct 0.24 (−6.4, 6.9)
indirect 0.3874 5.5 (−4.7, 16.)
network 0.92 (−4.5, 6.4)
−40
0
30

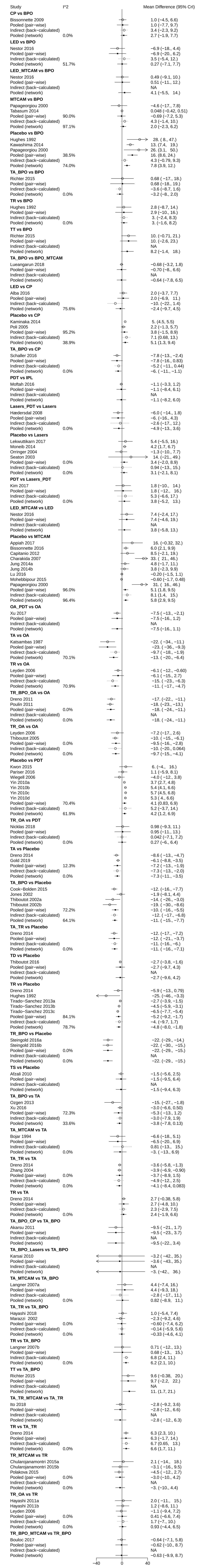

Study I^2 Mean Difference (95% CrI)
CP vs BPO
Bissonnette 2009 1.0 (−4.5, 6.6)
Pooled (pair−wise) 1.0 (−7.7, 9.7)
Indirect (back−calculated) 3.4 (−2.3, 9.2)
Pooled (network) 0.0% 2.7 (−1.9, 7.7)
LED vs BPO
Nestor 2016 −6.9 (−18., 4.4)
Pooled (pair−wise) −6.9 (−20., 6.2)
Indirect (back−calculated) 3.5 (−5.4, 12.)
Pooled (network) 51.7% 0.27 (−7.1, 7.7)
LED_MTCAM vs BPO
Nestor 2016 0.49 (−9.1, 10.)
Pooled (pair−wise) 0.51 (−11., 12.)
Indirect (back−calculated) NA
Pooled (network) 4.1 (−5.5, 14.)
MTCAM vs BPO
Papageorgiou 2000 −4.6 (−17., 7.8)
Tabasum 2014 0.048 (−0.42, 0.51)
Pooled (pair−wise) 90.0% −0.69 (−7.2, 5.3)
Indirect (back−calculated) 4.3 (−1.4, 10.)
Pooled (network) 97.1% 2.0 (−2.3, 6.2)
Placebo vs BPO
Hughes 1992 28. (8., 47.)
Kawashima 2014 13. (7.4, 19.)
Papageorgiou 2000 26. (3.1, 50.)
Pooled (pair−wise) 38.5% 16. (8.6, 24.)
Indirect (back−calculated) 4.3 (−0.79, 9.3)
Pooled (network) 74.0% 7.8 (3.9, 12.)
TA_BPO vs BPO
Richter 2015 0.68 (−17., 18.)
Pooled (pair−wise) 0.68 (−18., 19.)
Indirect (back−calculated) −3.6 (−8.7, 1.6)
Pooled (network) 0.0% −3.2 (−8., 2.0)
TR vs BPO
Hughes 1992 2.8 (−8.7, 14.)
Pooled (pair−wise) 2.9 (−10., 16.)
Indirect (back−calculated) 3. (−2.4, 8.3)
Pooled (network) 0.0% 3. (−1.6, 8.2)
TT vs BPO
Richter 2015 10. (−0.71, 21.)
Pooled (pair−wise) 10. (−2.6, 23.)
Indirect (back−calculated) NA
Pooled (network) 8.2 (−1.4, 18.)
TA_BPO vs BPO_MTCAM
Lueangarun 2018 −0.68 (−3.2, 1.8)
Pooled (pair−wise) −0.70 (−8., 6.6)
Indirect (back−calculated) NA
Pooled (network) −0.64 (−7.8, 6.5)
LED vs CP
Alba 2016 2.0 (−3.7, 7.7)
Pooled (pair−wise) 2.0 (−6.9, 11.)
Indirect (back−calculated) −10. (−22., 1.4)
Pooled (network) 75.6% −2.4 (−9.7, 4.5)
Placebo vs CP
Kaminaka 2014 5. (4.5, 5.5)
Poli 2005 2.2 (−1.3, 5.7)
Pooled (pair−wise) 95.2% 3.8 (−1.5, 8.9)
Indirect (back−calculated) 7.1 (0.68, 13.)
Pooled (network) 38.9% 5.1 (1.3, 9.4)
TA_BPO vs CP
Schaller 2016 −7.8 (−13., −2.4)
Pooled (pair−wise) −7.8 (−16., 0.83)
Indirect (back−calculated) −5.2 (−11., 0.44)
Pooled (network) 0.0% −6. (−11., −1.1)
PDT vs IPL
Moftah 2016 −1.1 (−3.3, 1.2)
Pooled (pair−wise) −1.1 (−8.4, 6.1)
Indirect (back−calculated) NA
Pooled (network) −1.1 (−8.2, 6.0)
Lasers_PDT vs Lasers
Hædersdal 2008 −6.0 (−14., 1.8)
Pooled (pair−wise) −6. (−16., 4.3)
Indirect (back−calculated) −2.6 (−17., 12.)
Pooled (network) 0.0% −4.9 (−13., 3.6)
Placebo vs Lasers
Lekwuttikarn 2017 5.4 (−5.5, 16.)
Moneib 2014 4.2 (1.7, 6.7)
Orringer 2004 −1.3 (−10., 7.7)
Seaton 2003 14. (−21., 49.)
Pooled (pair−wise) 0.0% 3.4 (−2.0, 8.9)
Indirect (back−calculated) 0.94 (−13., 15.)
Pooled (network) 0.0% 3.1 (−2.1, 8.1)
PDT vs Lasers_PDT
Kim 2017 1.8 (−10., 14.)
Pooled (pair−wise) 1.8 (−12., 16.)
Indirect (back−calculated) 5.3 (−6.6, 17.)
Pooled (network) 0.0% 3.8 (−5.2, 13.)
LED_MTCAM vs LED
Nestor 2016 7.4 (−2.4, 17.)
Pooled (pair−wise) 7.4 (−4.6, 19.)
Indirect (back−calculated) NA
Pooled (network) 3.8 (−5.8, 13.)
Placebo vs MTCAM
Appiah 2017 16. (−0.32, 32.)
Bissonnette 2016 6.0 (2.1, 9.9)
Capitanio 2012 8.5 (−2.1, 19.)
Charakida 2007 33. (21., 46.)
Jung 2014a 4.8 (−1.7, 11.)
Jung 2014b 3.8 (−2.3, 9.9)
Lu 2016 −0.20 (−1.5, 1.1)
Mohebbipour 2015 −0.60 (−1.7, 0.48)
Papageorgiou 2000 31. (16., 46.)
Pooled (pair−wise) 96.0% 5.1 (1.8, 9.5)
Indirect (back−calculated) 8.1 (1.4, 15.)
Pooled (network) 96.4% 5.8 (2.9, 9.5)
OA_PDT vs OA
Xu 2017 −7.5 (−13., −2.1)
Pooled (pair−wise) −7.5 (−16., 1.2)
Indirect (back−calculated) NA
Pooled (network) −7.5 (−16., 1.1)
TA vs OA
Katsambas 1987 −22. (−34., −11.)
Pooled (pair−wise) −23. (−36., −9.3)
Indirect (back−calculated) −9.7 (−18., −1.9)
Pooled (network) 70.1% −13. (−20., −6.4)
TR vs OA
Leyden 2006 −6.1 (−12., −0.60)
Pooled (pair−wise) −6.1 (−15., 2.7)
Indirect (back−calculated) −15. (−23., −6.3)
Pooled (network) 70.9% −11. (−17., −4.7)
TR_BPO_OA vs OA
Dreno 2011 −17. (−22., −11.)
Poulin 2011 −18. (−23., −13.)
Pooled (pair−wise) 0.0% −18. (−24., −11.)
Indirect (back−calculated) NA
Pooled (network) 0.0% −18. (−24., −11.)
TR_OA vs OA
Leyden 2006 −7.2 (−17., 2.6)
Thiboutot 2005 −10. (−15., −6.1)
Pooled (pair−wise) 0.0% −9.5 (−16., −2.8)
Indirect (back−calculated) −10. (−20., 0.064)
Pooled (network) 0.0% −9.7 (−15., −4.1)
Placebo vs PDT
Kwon 2015 6. (−4., 16.)
Pariser 2016 1.1 (−5.9, 8.1)
Wiegell 2006 −4.0 (−12., 3.8)
Yin 2010a 3.7 (2.7, 4.8)
Yin 2010b 5.4 (4.1, 6.6)
Yin 2010c 5.7 (4.5, 6.8)
Yin 2010d 5.3 (4., 6.6)
Pooled (pair−wise) 70.4% 4.1 (0.83, 6.9)
Indirect (back−calculated) 5.2 (−3.7, 14.)
Pooled (network) 61.9% 4.2 (1.2, 6.9)
TR_OA vs PDT
Nicklas 2018 0.98 (−9.3, 11.)
Pooled (pair−wise) 0.95 (−11., 13.)
Indirect (back−calculated) 0.042 (−7.1, 7.2)
Pooled (network) 0.0% 0.27 (−6., 6.4)
TA vs Placebo
Dreno 2014 −8.6 (−13., −4.7)
Gold 2019 −6.1 (−8.8, −3.5)
Pooled (pair−wise) 12.3% −7.2 (−13., −1.9)
Indirect (back−calculated) −7.3 (−13., −2.0)
Pooled (network) 0.0% −7.3 (−11., −3.5)
TA_BPO vs Placebo
Cook−Bolden 2015 −12. (−16., −7.7)
Jones 2002 −1.9 (−8.1, 4.4)
Thiboutot 2002a −14. (−26., −3.0)
Thiboutot 2002b −19. (−30., −8.6)
Pooled (pair−wise) 72.2% −10. (−16., −5.5)
Indirect (back−calculated) −12. (−17., −6.8)
Pooled (network) 64.1% −11. (−15., −7.7)
TA_TR vs Placebo
Dreno 2014 −12. (−17., −7.2)
Pooled (pair−wise) −12. (−21., −3.7)
Indirect (back−calculated) −11. (−16., −6.)
Pooled (network) 0.0% −11. (−16., −7.1)
TD vs Placebo
Thiboutot 2016 −2.7 (−3.8, −1.6)
Pooled (pair−wise) −2.7 (−9.7, 4.3)
Indirect (back−calculated) NA
Pooled (network) −2.7 (−9.6, 4.2)
TR vs Placebo
Dreno 2014 −5.9 (−13., 0.79)
Hughes 1992 −25. (−46., −3.3)
Tirado−Sanchez 2013a −2.7 (−3.9, −1.5)
Tirado−Sanchez 2013b −4.5 (−5.9, −3.1)
Tirado−Sanchez 2013c −6.5 (−7.7, −5.4)
Pooled (pair−wise) 84.1% −5.2 (−9.2, −1.7)
Indirect (back−calculated) −4. (−9.7, 1.7)
Pooled (network) 78.7% −4.8 (−8.0, −1.8)
TR_BPO vs Placebo
Steingold 2016a −22. (−29., −14.)
Steingold 2016b −22. (−30., −15.)
Pooled (pair−wise) 0.0% −22. (−29., −15.)
Indirect (back−calculated) NA
Pooled (network) 0.0% −22. (−29., −15.)
TS vs Placebo
Afzali 2010 −1.5 (−5.6, 2.5)
Pooled (pair−wise) −1.5 (−9.5, 6.4)
Indirect (back−calculated) NA
Pooled (network) −1.5 (−9.4, 6.3)
TA_BPO vs TA
Ozgen 2013 −15. (−27., −1.8)
Xu 2016 −3.0 (−6.6, 0.50)
Pooled (pair−wise) 72.3% −5.3 (−13., 1.2)
Indirect (back−calculated) −3.0 (−7.9, 1.9)
Pooled (network) 33.6% −3.8 (−7.8, 0.13)
TA_MTCAM vs TA
Bojar 1994 −6.6 (−18., 5.1)
Pooled (pair−wise) −6.5 (−20., 6.9)
Indirect (back−calculated) 0.81 (−13., 15.)
Pooled (network) 0.0% −3. (−13., 6.9)
TA_TR vs TA
Dreno 2014 −3.6 (−5.8, −1.3)
Zhang 2004 −3.9 (−6.9, −0.90)
Pooled (pair−wise) 0.0% −3.7 (−8.9, 1.5)
Indirect (back−calculated) −4.9 (−12., 2.5)
Pooled (network) 0.0% −4.1 (−8.4, 0.083)
TR vs TA
Dreno 2014 2.7 (−0.38, 5.8)
Pooled (pair−wise) 2.7 (−4.8, 10.)
Indirect (back−calculated) 2.3 (−2.9, 7.5)
Pooled (network) 0.0% 2.4 (−1.9, 6.6)
TA_BPO_CP vs TA_BPO
Akarsu 2011 −9.5 (−21., 1.7)
Pooled (pair−wise) −9.5 (−23., 3.7)
Indirect (back−calculated) NA
Pooled (network) −9.5 (−22., 3.4)
TA_BPO_Lasers vs TA_BPO
Karsai 2010 −3.2 (−42., 35.)
Pooled (pair−wise) −3.6 (−43., 35.)
Indirect (back−calculated) NA
Pooled (network) −3. (−42., 36.)
TA_MTCAM vs TA_BPO
Langner 2007a 4.4 (−7.4, 16.)
Pooled (pair−wise) 4.4 (−9.3, 18.)
Indirect (back−calculated) −2.8 (−17., 11.)
Pooled (network) 0.0% 0.82 (−8.9, 11.)
TA_TR vs TA_BPO
Hayashi 2018 1.0 (−5.4, 7.4)
Marazzi 2002 −2.3 (−9.2, 4.6)
Pooled (pair−wise) 0.0% −0.60 (−7.4, 6.2)
Indirect (back−calculated) −0.14 (−5.9, 5.6)
Pooled (network) 0.0% −0.33 (−4.6, 4.1)
TR vs TA_BPO
Langner 2007b 0.71 (−12., 13.)
Pooled (pair−wise) 0.68 (−13., 15.)
Indirect (back−calculated) 6.8 (2.4, 11.)
Pooled (network) 0.0% 6.2 (2.1, 10.)
TT vs TA_BPO
Richter 2015 9.6 (−0.38, 20.)
Pooled (pair−wise) 9.7 (−2.2, 22.)
Indirect (back−calculated) NA
Pooled (network) 11. (1.7, 21.)
TA_TR_MTCAM vs TA_TR
Ito 2018 −2.8 (−9.2, 3.6)
Pooled (pair−wise) −2.8 (−12., 6.6)
Indirect (back−calculated) NA
Pooled (network) −2.8 (−12., 6.3)
TR vs TA_TR
Dreno 2014 6.3 (2.3, 10.)
Pooled (pair−wise) 6.3 (−1.7, 14.)
Indirect (back−calculated) 6.7 (0.65, 13.)
Pooled (network) 0.0% 6.6 (1.7, 11.)
TR_MTCAM vs TR
Chularojanamontri 2015a 2.1 (−14., 18.)
Chularojanamontri 2015b −3.1 (−16., 9.5)
Polakova 2015 −4.5 (−12., 2.7)
Pooled (pair−wise) 0.0% −3.0 (−10., 4.2)
Indirect (back−calculated) NA
Pooled (network) 0.0% −3. (−10., 4.4)
TR_OA vs TR
Hayashi 2011a 2.0 (−11., 15.)
Hayashi 2011b 1.2 (−8.6, 11.)
Leyden 2006 −1.1 (−9.4, 7.2)
Pooled (pair−wise) 0.0% 0.41 (−6.6, 7.4)
Indirect (back−calculated) 1.7 (−7., 10.)
Pooled (network) 0.0% 0.93 (−4.4, 6.5)
TR_BPO_MTCAM vs TR_BPO
Bouloc 2017 −0.64 (−7.1, 5.8)
Pooled (pair−wise) −0.62 (−10., 8.7)
Indirect (back−calculated) NA
Pooled (network) −0.63 (−9.9, 8.7)
−40
0
40

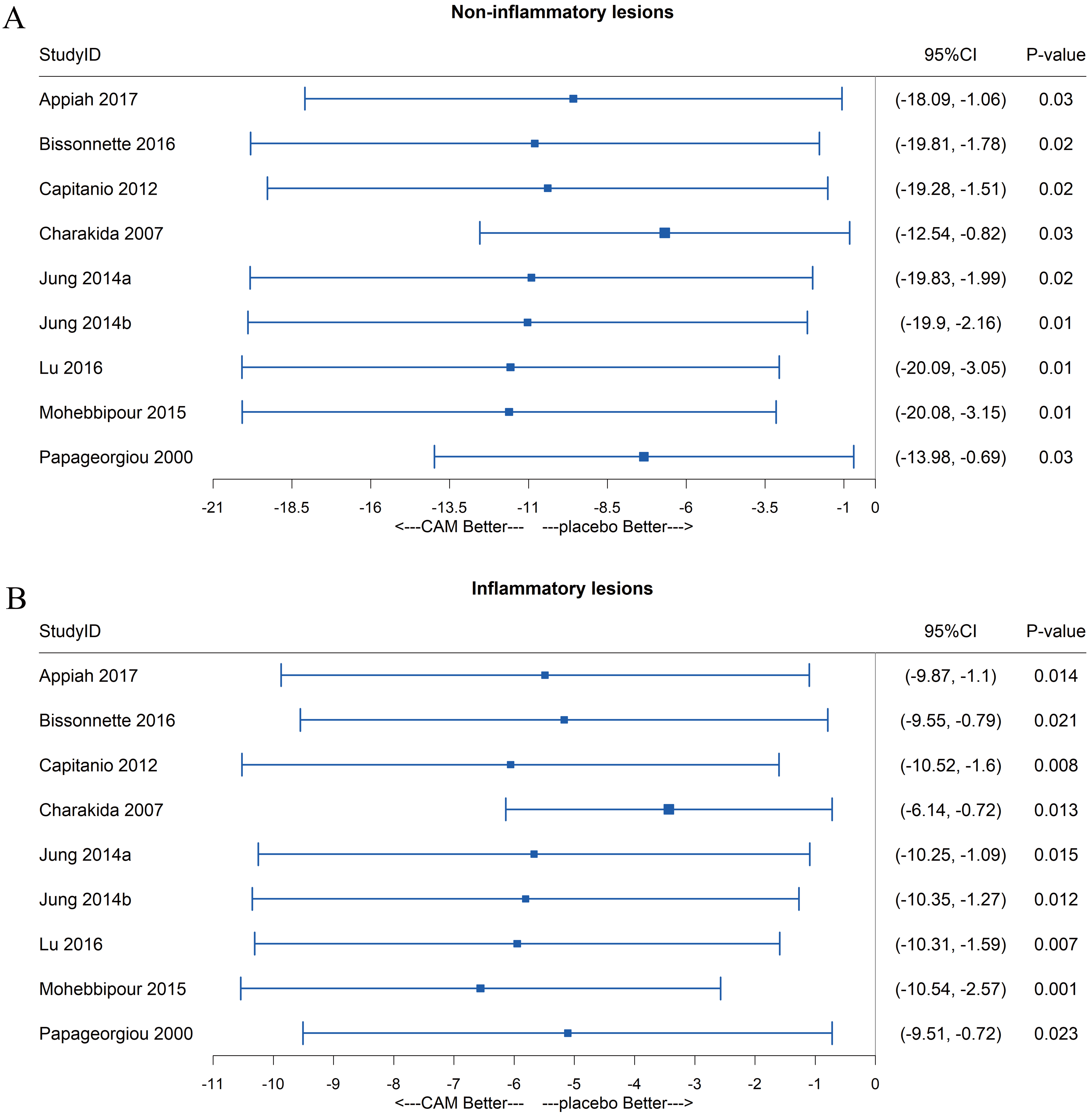
A
Non-inflammatory lesions
StudyID
95%CI
P-value
Appiah 2017
(-18.09, -1.06)
0.03
Bissonnette 2016
(-19.81, -1.78)
0.02
Capitanio 2012
(-19.28, -1.51)
0.02
Charakida 2007
(-12.54, -0.82)
0.03
Jung 2014a
(-19.83, -1.99)
0.02
Jung 2014b
(-19.9, -2.16)
0.01
Lu 2016
(-20.09, -3.05)
0.01
Mohebbipour 2015
(-20.08, -3.15)
0.01
Papageorgiou 2000
(-13.98, -0.69)
0.03
-21
-18.5
-16
-13.5
-11
-8.5
-6
-3.5
-1
0
<---CAM Better---
---placebo Better--->
B
Inflammatory lesions
StudyID
95%CI
P-value
Appiah 2017
(-9.87, -1.1)
0.014
Bissonnette 2016
(-9.55, -0.79)
0.021
Capitanio 2012
(-10.52, -1.6)
0.008
Charakida 2007
(-6.14, -0.72)
0.013
Jung 2014a
(-10.25, -1.09)
0.015
Jung 2014b
(-10.35, -1.27)
0.012
Lu 2016
(-10.31, -1.59)
0.007
Mohebbipour 2015
(-10.54, -2.57)
0.001
Papageorgiou 2000
(-9.51, -0.72)
0.023
-11
-10
-9
-8
-7
-6
-5
-4
-3
-2
-1
0
<---CAM Better---
---placebo Better--->

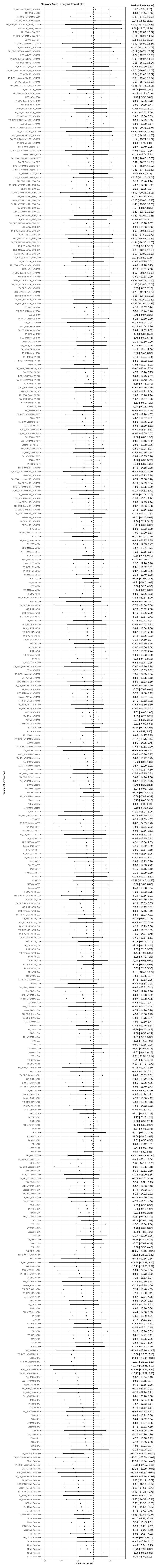

Network Meta-analysis Forest plot
Median [lower, upper]
Treatment comparison
Continuous Scale

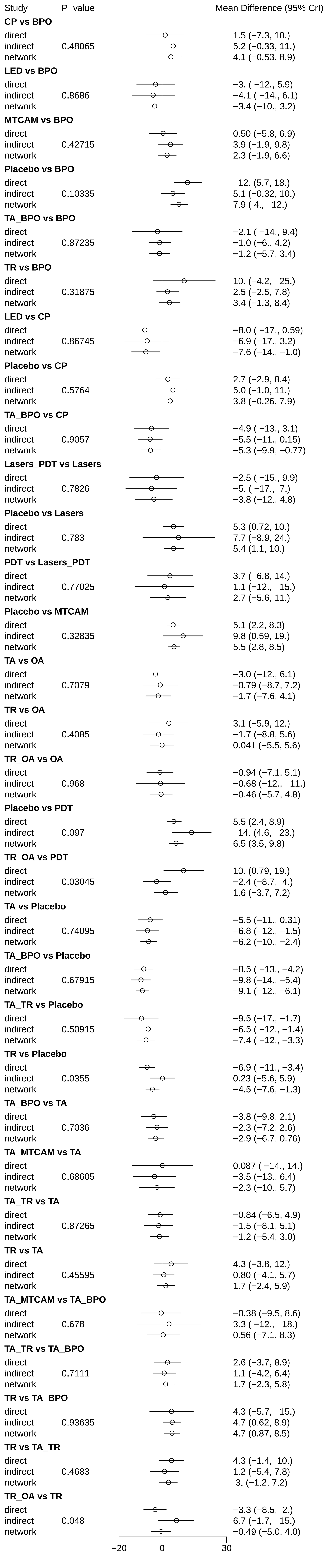
Study P−value Mean Difference (95% CrI)
CP vs BPO
direct 1.5 (−7.3, 10.)
indirect 0.48065 5.2 (−0.33, 11.)
network 4.1 (−0.53, 8.9)
LED vs BPO
direct −3. (−12., 5.9)
indirect 0.8686 −4.1 (−14., 6.1)
network −3.4 (−10., 3.2)
MTCAM vs BPO
direct 0.50 (−5.8, 6.9)
indirect 0.42715 3.9 (−1.9, 9.8)
network 2.3 (−1.9, 6.6)
Placebo vs BPO
direct 12. (5.7, 18.)
indirect 0.10335 5.1 (−0.32, 10.)
network 7.9 (4., 12.)
TA_BPO vs BPO
direct −2.1 (−14., 9.4)
indirect 0.87235 −1.0 (−6., 4.2)
network −1.2 (−5.7, 3.4)
TR vs BPO
direct 10. (−4.2, 25.)
indirect 0.31875 2.5 (−2.5, 7.8)
network 3.4 (−1.3, 8.4)
LED vs CP
direct −8.0 (−17., 0.59)
indirect 0.86745 −6.9 (−17., 3.2)
network −7.6 (−14., −1.0)
Placebo vs CP
direct 2.7 (−2.9, 8.4)
indirect 0.5764 5.0 (−1.0, 11.)
network 3.8 (−0.26, 7.9)
TA_BPO vs CP
direct −4.9 (−13., 3.1)
indirect 0.9057 −5.5 (−11., 0.15)
network −5.3 (−9.9, −0.77)
Lasers_PDT vs Lasers
direct −2.5 (−15., 9.9)
indirect 0.7826 −5. (−17., 7.)
network −3.8 (−12., 4.8)
Placebo vs Lasers
direct 5.3 (0.72, 10.)
indirect 0.783 7.7 (−8.9, 24.)
network 5.4 (1.1, 10.)
PDT vs Lasers_PDT
direct 3.7 (−6.8, 14.)
indirect 0.77025 1.1 (−12., 15.)
network 2.7 (−5.6, 11.)
Placebo vs MTCAM
direct 5.1 (2.2, 8.3)
indirect 0.32835 9.8 (0.59, 19.)
network 5.5 (2.8, 8.5)
TA vs OA
direct −3.0 (−12., 6.1)
indirect 0.7079 −0.79 (−8.7, 7.2)
network −1.7 (−7.6, 4.1)
TR vs OA
direct 3.1 (−5.9, 12.)
indirect 0.4085 −1.7 (−8.8, 5.6)
network 0.041 (−5.5, 5.6)
TR_OA vs OA
direct −0.94 (−7.1, 5.1)
indirect 0.968 −0.68 (−12., 11.)
network −0.46 (−5.7, 4.8)
Placebo vs PDT
direct 5.5 (2.4, 8.9)
indirect 0.097 14. (4.6, 23.)
network 6.5 (3.5, 9.8)
TR_OA vs PDT
direct 10. (0.79, 19.)
indirect 0.03045 −2.4 (−8.7, 4.)
network 1.6 (−3.7, 7.2)
TA vs Placebo
direct −5.5 (−11., 0.31)
indirect 0.74095 −6.8 (−12., −1.5)
network −6.2 (−10., −2.4)
TA_BPO vs Placebo
direct −8.5 (−13., −4.2)
indirect 0.67915 −9.8 (−14., −5.4)
network −9.1 (−12., −6.1)
TA_TR vs Placebo
direct −9.5 (−17., −1.7)
indirect 0.50915 −6.5 (−12., −1.4)
network −7.4 (−12., −3.3)
TR vs Placebo
direct −6.9 (−11., −3.4)
indirect 0.0355 0.23 (−5.6, 5.9)
network −4.5 (−7.6, −1.3)
TA_BPO vs TA
direct −3.8 (−9.8, 2.1)
indirect 0.7036 −2.3 (−7.2, 2.6)
network −2.9 (−6.7, 0.76)
TA_MTCAM vs TA
direct 0.087 (−14., 14.)
indirect 0.68605 −3.5 (−13., 6.4)
network −2.3 (−10., 5.7)
TA_TR vs TA
direct −0.84 (−6.5, 4.9)
indirect 0.87265 −1.5 (−8.1, 5.1)
network −1.2 (−5.4, 3.0)
TR vs TA
direct 4.3 (−3.8, 12.)
indirect 0.45595 0.80 (−4.1, 5.7)
network 1.7 (−2.4, 5.9)
TA_MTCAM vs TA_BPO
direct −0.38 (−9.5, 8.6)
indirect 0.678 3.3 (−12., 18.)
network 0.56 (−7.1, 8.3)
TA_TR vs TA_BPO
direct 2.6 (−3.7, 8.9)
indirect 0.7111 1.1 (−4.2, 6.4)
network 1.7 (−2.3, 5.8)
TR vs TA_BPO
direct 4.3 (−5.7, 15.)
indirect 0.93635 4.7 (0.62, 8.9)
network 4.7 (0.87, 8.5)
TR vs TA_TR
direct 4.3 (−1.4, 10.)
indirect 0.4683 1.2 (−5.4, 7.8)
network 3. (−1.2, 7.2)
TR_OA vs TR
direct −3.3 (−8.5, 2.)
indirect 0.048 6.7 (−1.7, 15.)
network −0.49 (−5.0, 4.0)
−20
0
30

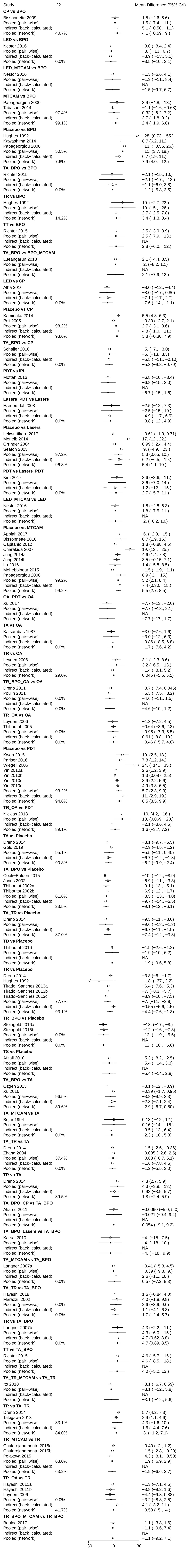
Study I^2 Mean Difference (95% CrI)
CP vs BPO
Bissonnette 2009 1.5 (−2.6, 5.6)
Pooled (pair−wise) 1.5 (−7.4, 11.)
Indirect (back−calculated) 5.1 (−0.50, 11.)
Pooled (network) 40.7% 4.1 (−0.59, 9.)
LED vs BPO
Nestor 2016 −3.0 (−8.4, 2.4)
Pooled (pair−wise) −3. (−13., 6.7)
Indirect (back−calculated) −3.9 (−13., 5.1)
Pooled (network) 0.0% −3.5 (−10., 3.1)
LED_MTCAM vs BPO
Nestor 2016 −1.3 (−6.6, 4.1)
Pooled (pair−wise) −1.3 (−11., 8.4)
Indirect (back−calculated) NA
Pooled (network) −1.5 (−9.7, 6.7)
MTCAM vs BPO
Papageorgiou 2000 3.9 (−4.8, 13.)
Tabasum 2014 −1.1 (−1.6, −0.68)
Pooled (pair−wise) 97.4% 0.32 (−6.2, 7.2)
Indirect (back−calculated) 3.7 (−1.8, 9.2)
Pooled (network) 99.1% 2.4 (−1.9, 6.6)
Placebo vs BPO
Hughes 1992 28. (0.73, 55.)
Kawashima 2014 8.7 (6.2, 11.)
Papageorgiou 2000 13. (−0.56, 26.)
Pooled (pair−wise) 50.5% 11. (3.7, 18.)
Indirect (back−calculated) 6.7 (1.9, 11.)
Pooled (network) 7.6% 7.9 (4.0, 12.)
TA_BPO vs BPO
Richter 2015 −2.1 (−15., 10.)
Pooled (pair−wise) −2.1 (−17., 13.)
Indirect (back−calculated) −1.1 (−6.0, 3.8)
Pooled (network) 0.0% −1.2 (−5.8, 3.5)
TR vs BPO
Hughes 1992 10. (−2.7, 23.)
Pooled (pair−wise) 10. (−5., 26.)
Indirect (back−calculated) 2.7 (−2.5, 7.8)
Pooled (network) 14.2% 3.4 (−1.3, 8.4)
TT vs BPO
Richter 2015 2.5 (−3.9, 8.9)
Pooled (pair−wise) 2.5 (−7.9, 13.)
Indirect (back−calculated) NA
Pooled (network) 2.8 (−6.0, 12.)
TA_BPO vs BPO_MTCAM
Lueangarun 2018 2.1 (−4.4, 8.5)
Pooled (pair−wise) 2. (−8.2, 12.)
Indirect (back−calculated) NA
Pooled (network) 2.1 (−7.9, 12.)
LED vs CP
Alba 2016 −8.0 (−12., −4.4)
Pooled (pair−wise) −8.0 (−17., 0.80)
Indirect (back−calculated) −7.1 (−17., 2.7)
Pooled (network) 0.0% −7.6 (−14., −1.1)
Placebo vs CP
Kaminaka 2014 5.5 (4.8, 6.3)
Poli 2005 −0.30 (−2.7, 2.1)
Pooled (pair−wise) 98.2% 2.7 (−3.1, 8.6)
Indirect (back−calculated) 4.8 (−1.0, 11.)
Pooled (network) 93.6% 3.8 (−0.30, 7.9)
TA_BPO vs CP
Schaller 2016 −5. (−7., −3.0)
Pooled (pair−wise) −5. (−13., 3.3)
Indirect (back−calculated) −5.5 (−11., −0.10)
Pooled (network) 0.0% −5.3 (−9.8, −0.79)
PDT vs IPL
Moftah 2016 −6.8 (−10., −3.4)
Pooled (pair−wise) −6.8 (−15., 2.0)
Indirect (back−calculated) NA
Pooled (network) −6.7 (−15., 1.6)
Lasers_PDT vs Lasers
Hædersdal 2008 −2.5 (−12., 7.3)
Pooled (pair−wise) −2.5 (−15., 10.)
Indirect (back−calculated) −4.9 (−17., 6.9)
Pooled (network) 0.0% −3.8 (−12., 4.9)
Placebo vs Lasers
Lekwuttikarn 2017 −0.61 (−1.9, 0.71)
Moneib 2014 17. (12., 22.)
Orringer 2004 0.99 (−2.4, 4.4)
Seaton 2003 9. (−4.9, 23.)
Pooled (pair−wise) 97.2% 5.3 (0.65, 10.)
Indirect (back−calculated) 6.2 (−6.5, 19.)
Pooled (network) 96.3% 5.4 (1.1, 10.)
PDT vs Lasers_PDT
Kim 2017 3.6 (−3.6, 11.)
Pooled (pair−wise) 3.6 (−7.0, 14.)
Indirect (back−calculated) 1.2 (−12., 15.)
Pooled (network) 0.0% 2.7 (−5.7, 11.)
LED_MTCAM vs LED
Nestor 2016 1.8 (−2.8, 6.3)
Pooled (pair−wise) 1.8 (−7.5, 11.)
Indirect (back−calculated) NA
Pooled (network) 2. (−6.2, 10.)
Placebo vs MTCAM
Appiah 2017 6. (−2.8, 15.)
Bissonnette 2016 8.7 (1.9, 15.)
Capitanio 2012 1.8 (−0.88, 4.5)
Charakida 2007 19. (13., 25.)
Jung 2014a 4.6 (1.4, 7.8)
Jung 2014b 3.5 (−0.15, 7.1)
Lu 2016 1.4 (−5.8, 8.5)
Mohebbipour 2015 −1.5 (−1.9, −1.1)
Papageorgiou 2000 8.9 (3., 15.)
Pooled (pair−wise) 99.2% 5.2 (2.1, 8.4)
Indirect (back−calculated) 7.4 (0.30, 15.)
Pooled (network) 99.2% 5.5 (2.7, 8.5)
OA_PDT vs OA
Xu 2017 −7.7 (−13., −2.0)
Pooled (pair−wise) −7.7 (−18., 2.1)
Indirect (back−calculated) NA
Pooled (network) −7.7 (−17., 1.7)
TA vs OA
Katsambas 1987 −3.0 (−7.6, 1.6)
Pooled (pair−wise) −3.0 (−12., 6.3)
Indirect (back−calculated) −0.86 (−8.5, 6.8)
Pooled (network) 0.0% −1.7 (−7.6, 4.2)
TR vs OA
Leyden 2006 3.1 (−2.3, 8.6)
Pooled (pair−wise) 3.2 (−6.5, 13.)
Indirect (back−calculated) −1.4 (−8.1, 5.2)
Pooled (network) 29.0% 0.046 (−5.5, 5.5)
TR_BPO_OA vs OA
Dreno 2011 −3.7 (−7.4, 0.045)
Poulin 2011 −5.3 (−7.5, −3.2)
Pooled (pair−wise) 0.0% −4.6 (−11., 1.5)
Indirect (back−calculated) NA
Pooled (network) 0.0% −4.6 (−10., 1.2)
TR_OA vs OA
Leyden 2006 −1.3 (−7.2, 4.5)
Thiboutot 2005 −0.64 (−3.6, 2.3)
Pooled (pair−wise) 0.0% −0.95 (−7.3, 5.5)
Indirect (back−calculated) 0.61 (−8.8, 10.)
Pooled (network) 0.0% −0.46 (−5.7, 4.8)
Placebo vs PDT
Kwon 2015 10. (2.5, 18.)
Pariser 2016 7.8 (1.2, 14.)
Wiegell 2006 24. (14., 35.)
Yin 2010a 2.6 (1.2, 3.9)
Yin 2010b 1.3 (0.087, 2.5)
Yin 2010c 3.9 (2.2, 5.6)
Yin 2010d 4.9 (3.3, 6.5)
Pooled (pair−wise) 93.2% 5.7 (2.3, 9.3)
Indirect (back−calculated) 11. (2.9, 19.)
Pooled (network) 94.6% 6.5 (3.5, 9.9)
TR_OA vs PDT
Nicklas 2018 10. (4.2, 16.)
Pooled (pair−wise) 10. (0.069, 20.)
Indirect (back−calculated) −2.1 (−8.6, 4.5)
Pooled (network) 89.1% 1.6 (−3.7, 7.2)
TA vs Placebo
Dreno 2014 −8.1 (−9.7, −6.5)
Gold 2019 −2.9 (−4.5, −1.2)
Pooled (pair−wise) 95.1% −5.5 (−11., 0.40)
Indirect (back−calculated) −6.7 (−12., −1.8)
Pooled (network) 90.8% −6.2 (−9.9, −2.4)
TA_BPO vs Placebo
Cook−Bolden 2015 −10. (−12., −8.9)
Jones 2002 −6.9 (−11., −3.3)
Thiboutot 2002a −9.1 (−13., −5.1)
Thiboutot 2002b −6.9 (−12., −1.7)
Pooled (pair−wise) 61.6% −8.5 (−13., −4.0)
Indirect (back−calculated) −9.7 (−14., −5.5)
Pooled (network) 23.5% −9.1 (−12., −6.1)
TA_TR vs Placebo
Dreno 2014 −9.5 (−11., −8.0)
Pooled (pair−wise) −9.6 (−18., −1.3)
Indirect (back−calculated) −6.7 (−11., −1.9)
Pooled (network) 87.0% −7.4 (−12., −3.3)
TD vs Placebo
Thiboutot 2016 −1.9 (−2.6, −1.2)
Pooled (pair−wise) −1.9 (−10., 6.2)
Indirect (back−calculated) NA
Pooled (network) −1.9 (−9.6, 5.8)
TR vs Placebo
Dreno 2014 −3.8 (−6., −1.7)
Hughes 1992 −18. (−37., 2.2)
Tirado−Sanchez 2013a −6.4 (−7.6, −5.3)
Tirado−Sanchez 2013b −7. (−8.3, −5.7)
Tirado−Sanchez 2013c −8.9 (−10., −7.5)
Pooled (pair−wise) 77.7% −7. (−11., −2.9)
Indirect (back−calculated) −0.55 (−5.6, 4.5)
Pooled (network) 93.1% −4.4 (−7.6, −1.3)
TR_BPO vs Placebo
Steingold 2016a −13. (−17., −8.)
Steingold 2016b −12. (−16., −7.3)
Pooled (pair−wise) 0.0% −12. (−19., −5.6)
Indirect (back−calculated) NA
Pooled (network) 0.0% −12. (−18., −5.8)
TS vs Placebo
Afzali 2010 −5.3 (−8.2, −2.5)
Pooled (pair−wise) −5.4 (−14., 3.3)
Indirect (back−calculated) NA
Pooled (network) −5.4 (−14., 2.8)
TA_BPO vs TA
Ozgen 2013 −8.1 (−12., −3.9)
Xu 2016 −0.39 (−1.7, 0.95)
Pooled (pair−wise) 96.5% −3.8 (−9.9, 2.3)
Indirect (back−calculated) −2.3 (−7.1, 2.4)
Pooled (network) 89.6% −2.9 (−6.7, 0.80)
TA_MTCAM vs TA
Bojar 1994 0.18 (−12., 12.)
Pooled (pair−wise) 0.16 (−14., 15.)
Indirect (back−calculated) −3.5 (−13., 6.4)
Pooled (network) 0.0% −2.3 (−10., 5.8)
TA_TR vs TA
Dreno 2014 −1.5 (−2.6, −0.36)
Zhang 2004 −0.085 (−2.6, 2.5)
Pooled (pair−wise) 37.4% −0.83 (−6.7, 5.1)
Indirect (back−calculated) −1.6 (−7.8, 4.6)
Pooled (network) 0.0% −1.2 (−5.5, 3.0)
TR vs TA
Dreno 2014 4.3 (2.7, 5.9)
Pooled (pair−wise) 4.3 (−3.9, 13.)
Indirect (back−calculated) 0.92 (−3.9, 5.7)
Pooled (network) 89.5% 1.8 (−2.4, 5.9)
TA_BPO_CP vs TA_BPO
Akarsu 2011 −0.0090 (−5.0, 5.0)
Pooled (pair−wise) −0.021 (−9.4, 9.4)
Indirect (back−calculated) NA
Pooled (network) 0.054 (−9.1, 9.2)
TA_BPO_Lasers vs TA_BPO
Karsai 2010 −4. (−15., 7.5)
Pooled (pair−wise) −4. (−18., 10.)
Indirect (back−calculated) NA
Pooled (network) −4. (−18., 9.9)
TA_MTCAM vs TA_BPO
Langner 2007a −0.41 (−5.3, 4.5)
Pooled (pair−wise) −0.39 (−9.8, 9.)
Indirect (back−calculated) 2.6 (−11., 16.)
Pooled (network) 0.0% 0.57 (−7.2, 8.3)
TA_TR vs TA_BPO
Hayashi 2018 1.6 (−0.84, 4.0)
Marazzi 2002 4.0 (−1.8, 9.8)
Pooled (pair−wise) 0.0% 2.6 (−3.9, 9.0)
Indirect (back−calculated) 1.1 (−4.1, 6.3)
Pooled (network) 0.0% 1.7 (−2.4, 5.7)
TR vs TA_BPO
Langner 2007b 4.3 (−2.2, 11.)
Pooled (pair−wise) 4.3 (−6.0, 15.)
Indirect (back−calculated) 4.7 (0.62, 8.8)
Pooled (network) 0.0% 4.7 (0.89, 8.5)
TT vs TA_BPO
Richter 2015 4.6 (−5.7, 15.)
Pooled (pair−wise) 4.6 (−8.5, 18.)
Indirect (back−calculated) NA
Pooled (network) 4.0 (−5.2, 13.)
TA_TR_MTCAM vs TA_TR
Ito 2018 −3.1 (−6.7, 0.59)
Pooled (pair−wise) −3.1 (−12., 5.8)
Indirect (back−calculated) NA
Pooled (network) −3.1 (−12., 5.6)
TR vs TA_TR
Dreno 2014 5.7 (4.2, 7.3)
Takigawa 2013 2.9 (1.1, 4.6)
Pooled (pair−wise) 83.1% 4.3 (−1.6, 10.)
Indirect (back−calculated) 1.6 (−4.4, 7.6)
Pooled (network) 84.0% 3. (−1.2, 7.1)
TR_MTCAM vs TR
Chularojanamontri 2015a −0.40 (−2., 1.2)
Chularojanamontri 2015b −1.5 (−2.8, −0.20)
Polakova 2015 −4.3 (−8.1, −0.50)
Pooled (pair−wise) 63.0% −1.9 (−6.9, 2.9)
Indirect (back−calculated) NA
Pooled (network) 63.2% −1.9 (−6.6, 2.7)
TR_OA vs TR
Hayashi 2011a −1.3 (−7.1, 4.5)
Hayashi 2011b −3.8 (−9.2, 1.6)
Leyden 2006 −4.4 (−9.8, 0.88)
Pooled (pair−wise) 0.0% −3.2 (−8.8, 2.5)
Indirect (back−calculated) 4.1 (−3.2, 11.)
Pooled (network) 41.7% −0.50 (−5., 4.)
TR_BPO_MTCAM vs TR_BPO
Bouloc 2017 −1.1 (−3.8, 1.6)
Pooled (pair−wise) −1.1 (−9.6, 7.4)
Indirect (back−calculated) NA
Pooled (network) −1.1 (−9.2, 7.1)
−30
0
30

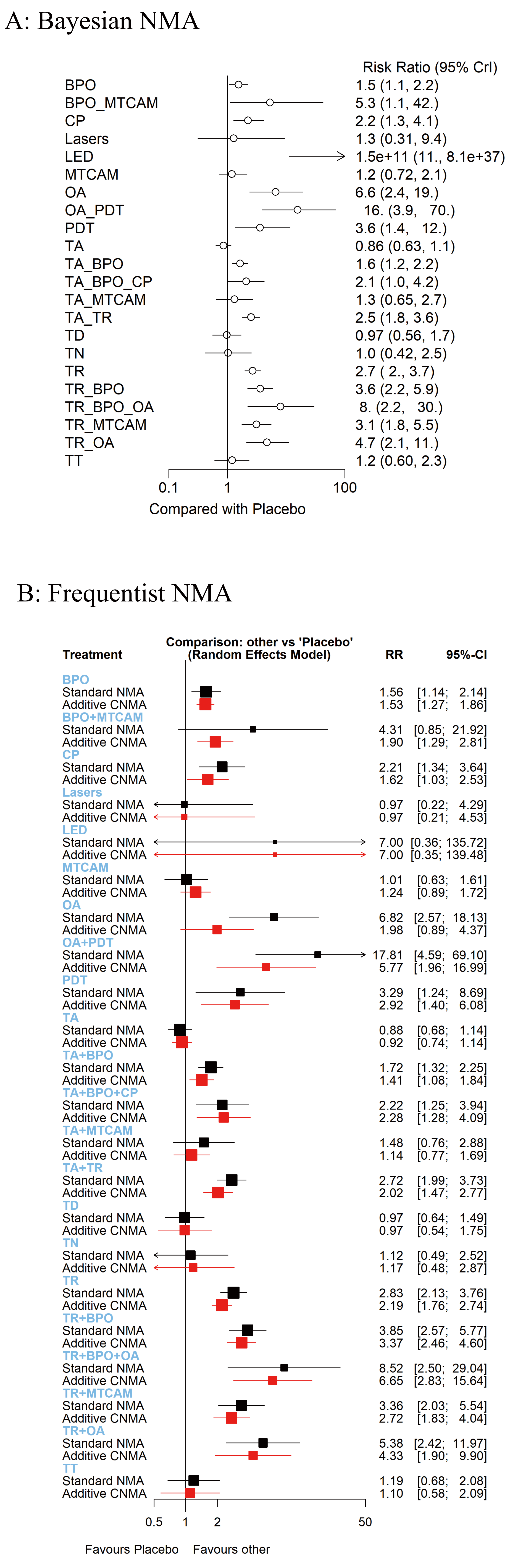
A: Bayesian NMA
Risk Ratio (95% CrI)
BPO 1.5 (1.1, 2.2)
BPO_MTCAM 5.3 (1.1, 42.)
CP 2.2 (1.3, 4.1)
Lasers 1.3 (0.31, 9.4)
LED 1.5e+11 (11., 8.1e+37)
MTCAM 1.2 (0.72, 2.1)
OA 6.6 (2.4, 19.)
OA_PDT 16. (3.9, 70.)
PDT 3.6 (1.4, 12.)
TA 0.86 (0.63, 1.1)
TA_BPO 1.6 (1.2, 2.2)
TA_BPO_CP 2.1 (1.0, 4.2)
TA_MTCAM 1.3 (0.65, 2.7)
TA_TR 2.5 (1.8, 3.6)
TD 0.97 (0.56, 1.7)
TN 1.0 (0.42, 2.5)
TR 2.7 (2., 3.7)
TR_BPO 3.6 (2.2, 5.9)
TR_BPO_OA 8. (2.2, 30.)
TR_MTCAM 3.1 (1.8, 5.5)
TR_OA 4.7 (2.1, 11.)
TT 1.2 (0.60, 2.3)
0.1
1
100
Compared with Placebo
B: Frequentist NMA
Treatment
Comparison: other vs 'Placebo'
(Random Effects Model)
RR
95%-CI
BPO
Standard NMA 1.56 [1.14; 2.14]
Additive CNMA 1.53 [1.27; 1.86]
BPO+MTCAM
Standard NMA 4.31 [0.85; 21.92]
Additive CNMA 1.90 [1.29; 2.81]
CP
Standard NMA 2.21 [1.34; 3.64]
Additive CNMA 1.62 [1.03; 2.53]
Lasers
Standard NMA 0.97 [0.22; 4.29]
Additive CNMA 0.97 [0.21; 4.53]
LED
Standard NMA 7.00 [0.36; 135.72]
Additive CNMA 7.00 [0.35; 139.48]
MTCAM
Standard NMA 1.01 [0.63; 1.61]
Additive CNMA 1.24 [0.89; 1.72]
OA
Standard NMA 6.82 [2.57; 18.13]
Additive CNMA 1.98 [0.89; 4.37]
OA+PDT
Standard NMA 17.81 [4.59; 69.10]
Additive CNMA 5.77 [1.96; 16.99]
PDT
Standard NMA 3.29 [1.24; 8.69]
Additive CNMA 2.92 [1.40; 6.08]
TA
Standard NMA 0.88 [0.68; 1.14]
Additive CNMA 0.92 [0.74; 1.14]
TA+BPO
Standard NMA 1.72 [1.32; 2.25]
Additive CNMA 1.41 [1.08; 1.84]
TA+BPO+CP
Standard NMA 2.22 [1.25; 3.94]
Additive CNMA 2.28 [1.28; 4.09]
TA+MTCAM
Standard NMA 1.48 [0.76; 2.88]
Additive CNMA 1.14 [0.77; 1.69]
TA+TR
Standard NMA 2.72 [1.99; 3.73]
Additive CNMA 2.02 [1.47; 2.77]
TD
Standard NMA 0.97 [0.64; 1.49]
Additive CNMA 0.97 [0.54; 1.75]
TN
Standard NMA 1.12 [0.49; 2.52]
Additive CNMA 1.17 [0.48; 2.87]
TR
Standard NMA 2.83 [2.13; 3.76]
Additive CNMA 2.19 [1.76; 2.74]
TR+BPO
Standard NMA 3.85 [2.57; 5.77]
Additive CNMA 3.37 [2.46; 4.60]
TR+BPO+OA
Standard NMA 8.52 [2.50; 29.04]
Additive CNMA 6.65 [2.83; 15.64]
TR+MTCAM
Standard NMA 3.36 [2.03; 5.54]
Additive CNMA 2.72 [1.83; 4.04]
TR+OA
Standard NMA 5.38 [2.42; 11.97]
Additive CNMA 4.33 [1.90; 9.90]
TT
Standard NMA 1.19 [0.68; 2.08]
Additive CNMA 1.10 [0.58; 2.09]
0.5
1
2
50
Favours Placebo
Favours other

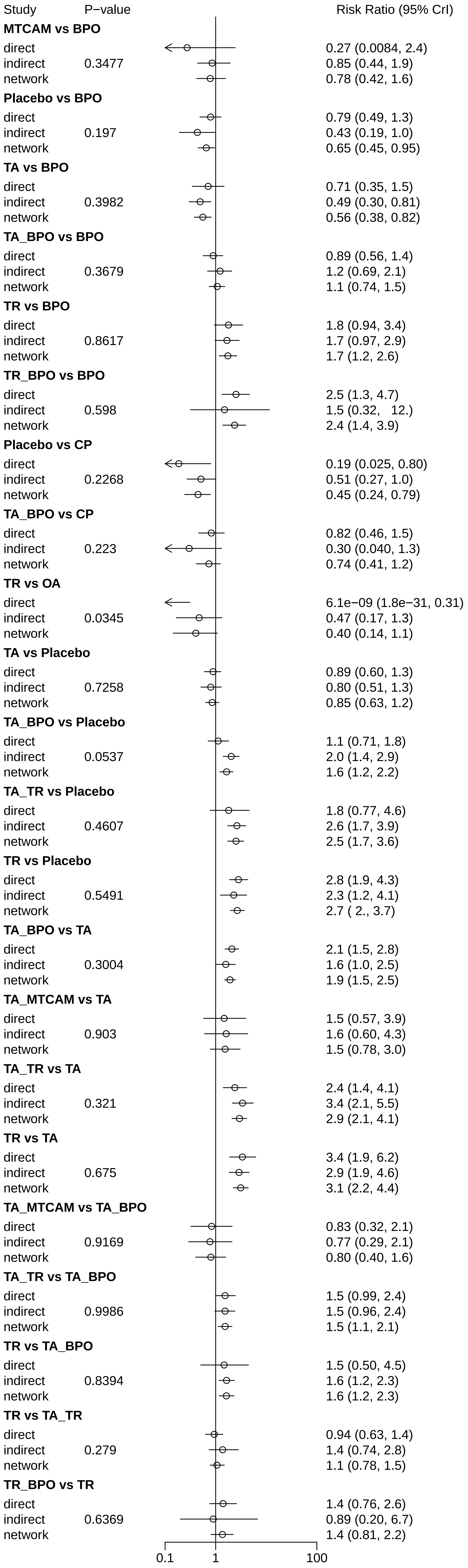

Study P−value Risk Ratio (95% CrI)
MTCAM vs BPO
direct 0.27 (0.0084, 2.4)
indirect 0.3477 0.85 (0.44, 1.9)
network 0.78 (0.42, 1.6)
Placebo vs BPO
direct 0.79 (0.49, 1.3)
indirect 0.197 0.43 (0.19, 1.0)
network 0.65 (0.45, 0.95)
TA vs BPO
direct 0.71 (0.35, 1.5)
indirect 0.3982 0.49 (0.30, 0.81)
network 0.56 (0.38, 0.82)
TA_BPO vs BPO
direct 0.89 (0.56, 1.4)
indirect 0.3679 1.2 (0.69, 2.1)
network 1.1 (0.74, 1.5)
TR vs BPO
direct 1.8 (0.94, 3.4)
indirect 0.8617 1.7 (0.97, 2.9)
network 1.7 (1.2, 2.6)
TR_BPO vs BPO
direct 2.5 (1.3, 4.7)
indirect 0.598 1.5 (0.32, 12.)
network 2.4 (1.4, 3.9)
Placebo vs CP
direct 0.19 (0.025, 0.80)
indirect 0.2268 0.51 (0.27, 1.0)
network 0.45 (0.24, 0.79)
TA_BPO vs CP
direct 0.82 (0.46, 1.5)
indirect 0.223 0.30 (0.040, 1.3)
network 0.74 (0.41, 1.2)
TR vs OA
direct 6.1e−09 (1.8e−31, 0.31)
indirect 0.0345 0.47 (0.17, 1.3)
network 0.40 (0.14, 1.1)
TA vs Placebo
direct 0.89 (0.60, 1.3)
indirect 0.7258 0.80 (0.51, 1.3)
network 0.85 (0.63, 1.2)
TA_BPO vs Placebo
direct 1.1 (0.71, 1.8)
indirect 0.0537 2.0 (1.4, 2.9)
network 1.6 (1.2, 2.2)
TA_TR vs Placebo
direct 1.8 (0.77, 4.6)
indirect 0.4607 2.6 (1.7, 3.9)
network 2.5 (1.7, 3.6)
TR vs Placebo
direct 2.8 (1.9, 4.3)
indirect 0.5491 2.3 (1.2, 4.1)
network 2.7 (2., 3.7)
TA_BPO vs TA
direct 2.1 (1.5, 2.8)
indirect 0.3004 1.6 (1.0, 2.5)
network 1.9 (1.5, 2.5)
TA_MTCAM vs TA
direct 1.5 (0.57, 3.9)
indirect 0.903 1.6 (0.60, 4.3)
network 1.5 (0.78, 3.0)
TA_TR vs TA
direct 2.4 (1.4, 4.1)
indirect 0.321 3.4 (2.1, 5.5)
network 2.9 (2.1, 4.1)
TR vs TA
direct 3.4 (1.9, 6.2)
indirect 0.675 2.9 (1.9, 4.6)
network 3.1 (2.2, 4.4)
TA_MTCAM vs TA_BPO
direct 0.83 (0.32, 2.1)
indirect 0.9169 0.77 (0.29, 2.1)
network 0.80 (0.40, 1.6)
TA_TR vs TA_BPO
direct 1.5 (0.99, 2.4)
indirect 0.9986 1.5 (0.96, 2.4)
network 1.5 (1.1, 2.1)
TR vs TA_BPO
direct 1.5 (0.50, 4.5)
indirect 0.8394 1.6 (1.2, 2.3)
network 1.6 (1.2, 2.3)
TR vs TA_TR
direct 0.94 (0.63, 1.4)
indirect 0.279 1.4 (0.74, 2.8)
network 1.1 (0.78, 1.5)
TR_BPO vs TR
direct 1.4 (0.76, 2.6)
indirect 0.6369 0.89 (0.20, 6.7)
network 1.4 (0.81, 2.2)
0.1
1
100

A: Funnel plot of non-inflammatory lesions

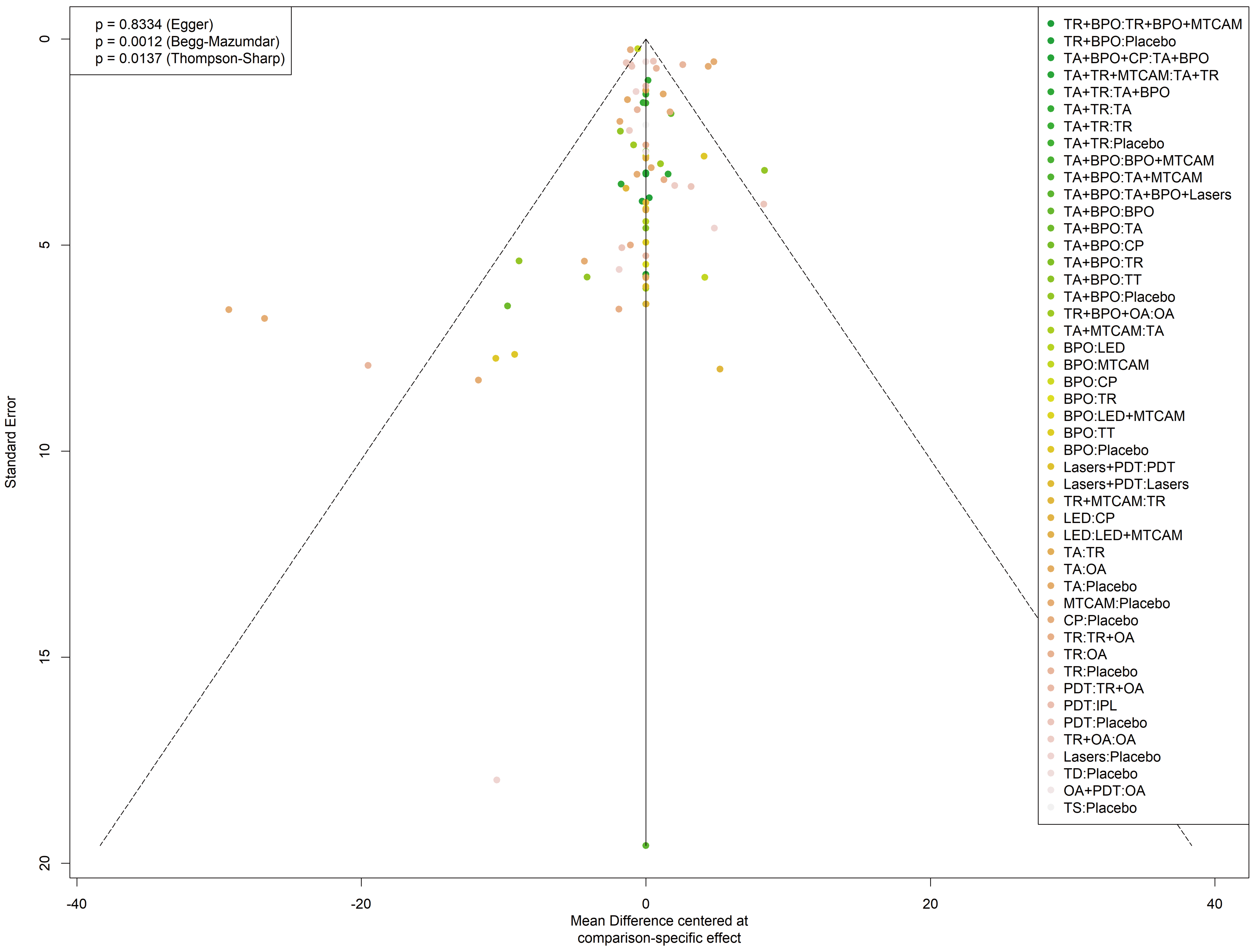

B: Funnel plot of inflammatory lesions

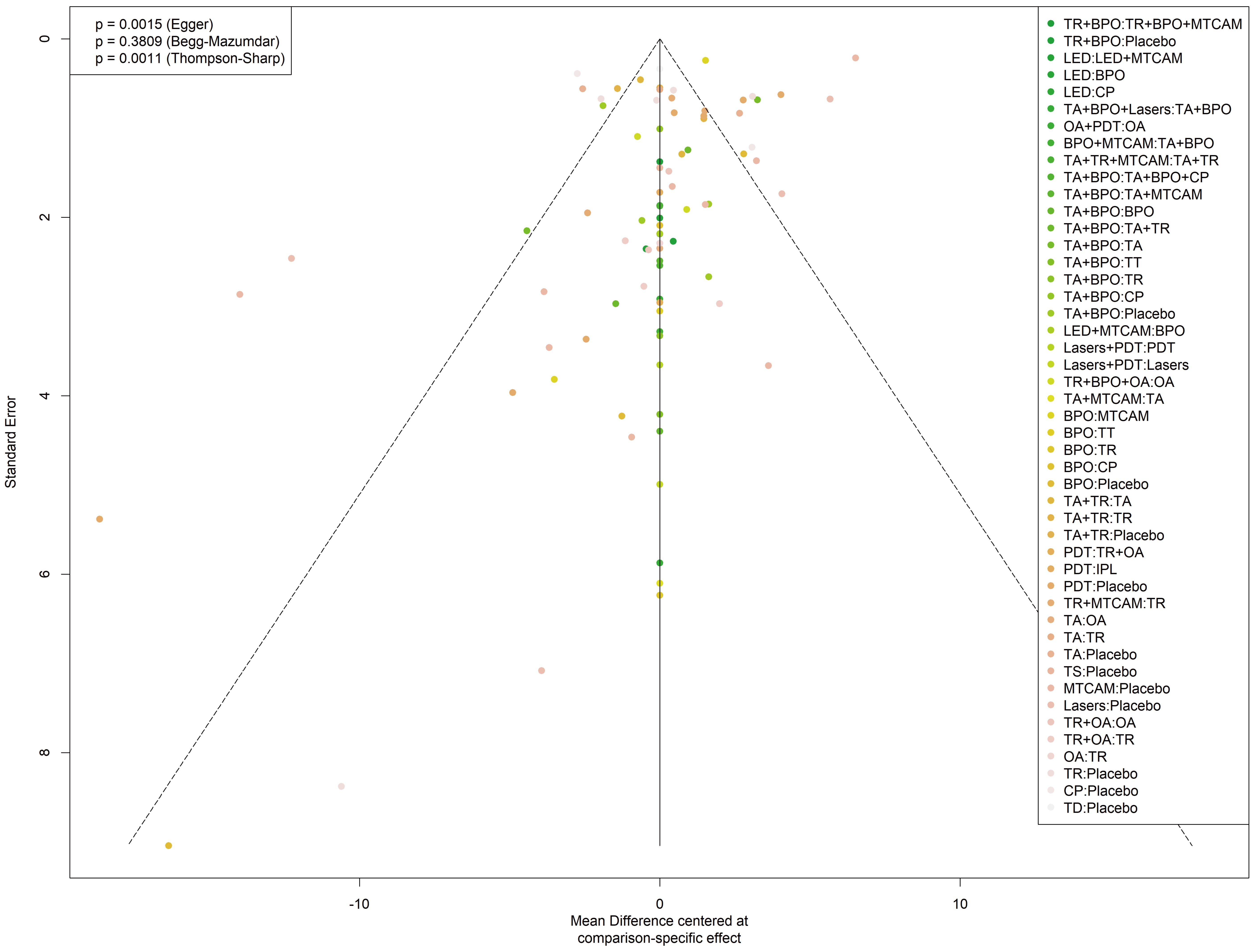

C: Funnel plot of adverse effects

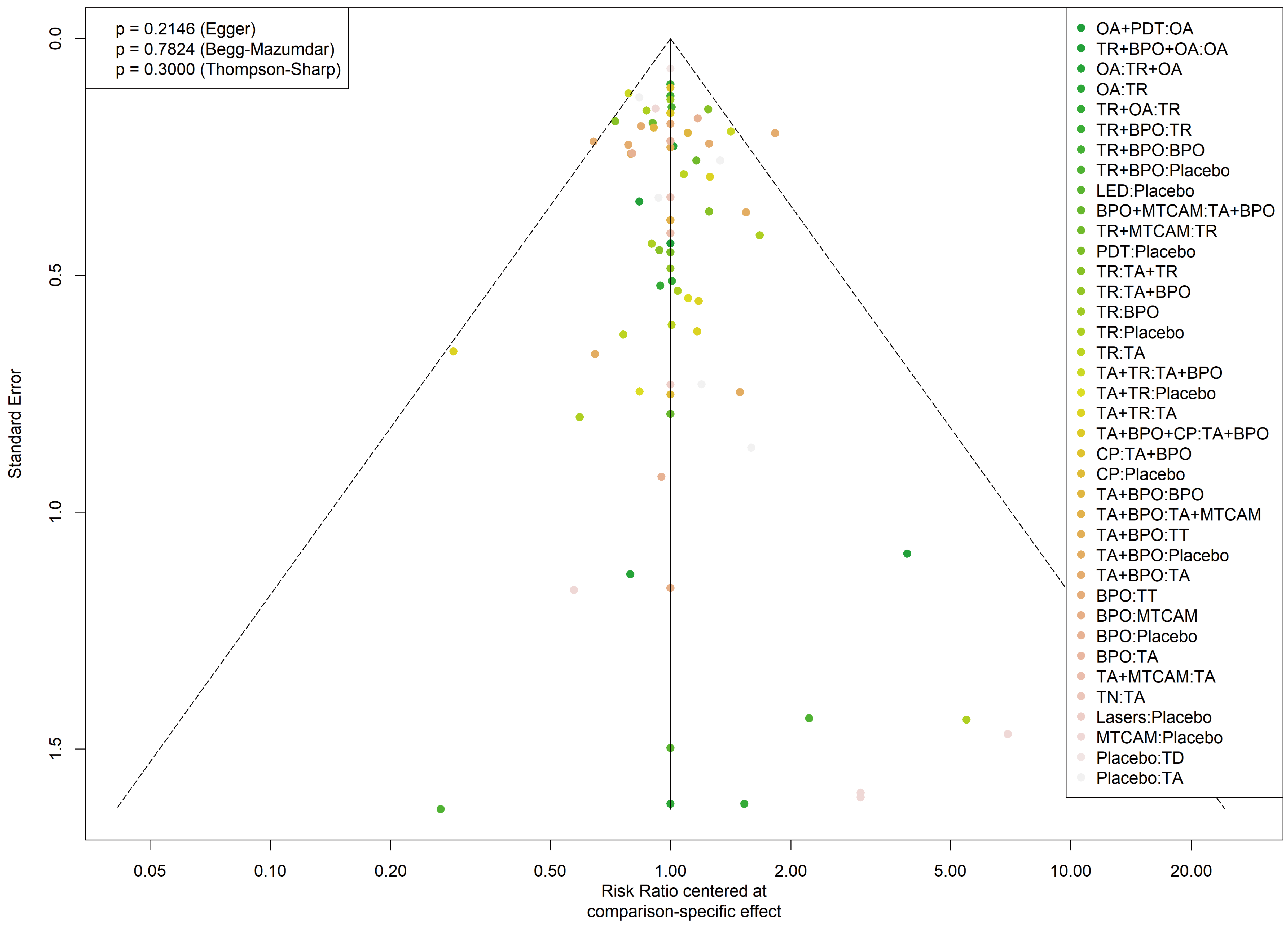

Supplement: Supplementary file 1 [file image1.pdf]
