# Supplementary material for: Comparative Efficacy of Pharmacological and Nonpharmacological Interventions for Acne Vulgaris: A Network Meta-Analysis
Source: Front Pharmacol. 2020 Nov 26;11:592075. doi: 10.3389/fphar.2020.592075 (PMC7729523; doi:10.3389/fphar.2020.592075)
Supplement: Supplementary file 2 [file datasheet1.docx]

**S1 Appendix**

Table of Contents

[Table 1 Included studies 2](#_Toc52902052)

[Table 2 Risk of bias 13](#_Toc52902053)

[Table 3 Meta-regression of non-inflammatory lesions reduction 19](#_Toc52902054)

[Table 4 SUCRA of non-inflammatory lesions reduction after adjustment 20](#_Toc52902055)

[Table 5 Meta-regression of inflammatory lesions reduction 22](#_Toc52902056)

[Table 6 SUCRA of inflammatory lesions reduction after adjustment 23](#_Toc52902057)

[File 1 Protocol amendments 25](#_Toc52902058)

[File 2 Search strategy 26](#_Toc52902059)

[File 3 Complete statistical analysis 28](#_Toc52902060)

[File 4 Choice of Prior Distributions 31](#_Toc52902061)

[File 5 Reference 32](#_Toc52902062)

# Table 1 Included studies

| Author (year) | Country | No. of sites | Age (mean) | Women | No. of patients | Interventions (per arm) | Duration of treatment (weeks) |
| --- | --- | --- | --- | --- | --- | --- | --- |
| Afzali 2010 | Iran | 1 | 21.76 | 0.82 | 62 | 1. 5% topical spironolactone gel  2. placebo | 6 |
| Akarsu 2011 | Turkey | 1 | 19 | 0.73 | 49 | 1. clindamycin 1%+ benzoyl peroxide 5% + salicylic acid 3%  2. clindamycin 1%+ benzoyl peroxide 5% | 12 |
| Alba 2016 | Brazil | 1 | 15.6 | 0.59 | 22 | 1. salicylic acid 10 %  2. blue light (a wavelength of about 475 nm) + red light (a wavelength of about 650 nm) | 10 |
| Appiah 2017 | UK | 1 | 30 | 0.71 | 21 | 1. Eladi Keram (Ayurvedic medicine)  2. vehicle control (coconut oil) | 4 |
| Bissonnette 2009 | Canada, France | 2 | 25.7 | 0.65 | 80 | 1. lipophillic hydroxy acid derivative of salicylic acid  2. benzoyl peroxide 5% | 12 |
| Bissonnette 2016 | Canada, USA | 12 | 25.2 | 0.65 | 108 | 1. Olumacostat glasaretil (a novel topical sebum inhibitor)  2. placebo | 12 |
| Bojar 1994 | UK | 1 | 19.15 | 0.33 | 45 | 1. topical 4% erythromycin  2. topical 4% erythromycin + 1.2% zinc acetate | 12 |
| Bouloc 2017 | France, Spain, Poland | 2 | 29.5 | 1 | 300 | 1. a standard non-comedogenic emollient (containing water, glycerin, stearic acid, butylene glycol, cyclo hexasiloxane) + adapalene/benzoyl peroxide  2. vehicle control + adapalene/benzoyl peroxide | 13 |
| Capitanio 2012 | Italy, UK | 1 | 18.1 | 0 | 60 | 1. a seaweed-derived oligosaccharide complexed with 0.1% zinc pyrrolidone  2. vehicle control | 8 |
| Charakida 2007 | UK | 1 | 25.75 | 0.55 | 40 | 1. triethyl citrate and ethyl linoleate  2. placebo | 12 |
| Chularojanamontri 2015 | Thailand | 1 | 27.5 | 0.55 | 80 | 1. adapalene gel  2. adapalene gel + moisturizer (containing licochalcone A, L-carnitine and 1,2-decanediol)  3. adapalene gel + placebo | 8 |
| Cook-Bolden 2015 | USA | >1* | 15 | 0.398 | 289 | 1. clindamycin phosphate 1.2% /benzoyl peroxide 3.75%  2. vehicle | 12 |
| Dreno 2011 | France, Germany, Brazil, Mexico, Belgium, Sweden, Australia, Poland, Italy | 32 | 18.9 | 0.45 | 378 | 1. adapalene 0.1% + BPO 2.5% + oral lymecycline  2. oral lymecycline + vehicle gel | 12 |
| Dreno 2014 | France, Italy, Germany, UK, Spain | >1* | 19.3 | 0.513 | 4550 | 1. Clindamycin phosphate 1.2%/tretinoin 0.025%  2. clindamycin phosphate 1.2%  3. tretinoin  4. Vehicle | 12 |
| Fonseca 1995 | Spain | 8 | 19 | 0.61 | 227 | 1. tretinoin + erythromycin lauryl sulphate  2. erythromycin lauryl sulphate  3. tretinoin | 10 |
| Glass 1999 | UK | 1 | 18.55 | 0.62 | 60 | 1. 0.05% isotretinoin and 2% erythromycin  2. 0.05% isotretinoin gel  3. 2% erythromycin  4. placebo | 12 |
| Gold 2016 | USA | 31 | 19.3 | 0.523 | 503 | 1. adapalene 0.3 %/benzoyl peroxide 2.5 %  2. adapalene 0.1 %/benzoyl peroxide 2.5 %  3. vehicle | 12 |
| Gold 2019 | USA | 30 | 20.5 | 0.58 | 961 | 1. FMX101 4% topical minocycline foam  2. vehicle | 12 |
| Hædersdal 2008 | Denmark | 1 | 18 | 0.66 | 30 | 1. long-pulsed dye laser  2. long-pulsed dye laser + assisted photodynamic therapy with methyl-aminolevulinic acid | 12 |
| Habbema 1989 | Netherlands,  Belgium | 2 | NA | NA | 122 | 1. 4% erythromycin + zinc  2. 2% erythromycin | 12 |
| Hajheydari 2013 | Iran | 1 | 23.5 | 1 | 60 | 1. tretinoin + aloe gel  2. tretinoin + placebo | 8 |
| Hayashi 2011 | Japan | 25 | 24 | 0.68 | 149 | 1. monotherapy with topical adapalene for 4 weeks  2. combination therapy with topical adapalene and oral faropenem for 2 weeks followed by topical adapalene alone for 2 weeks  3. combination therapy with topical adapalene and oral faropenem for 4 weeks | 4 |
| Hayashi 2018 | Japan, UK | 15 | 20.05 | 0.59 | 333 | 1. clindamycin phosphate + 3% benzoyl peroxide  2. adapalene + clindamycin phosphate | 12 |
| Hughes 1992 | UK | 1 | 18.7 | 0.4 | 77 | 1. isotretinoin gel 0.05%  2. benzoyl peroxide gel 5%  3. plaeebo | 12 |
| Ito 2018 | Japan | 8 | 24.1 | 0.59 | 52 | 1. Keigairengyoto (Japanese traditional medication) + 0.1% adapalene gel + antimicrobial agents (clindamycin gel or nadifloxacin cream)  2. 0.1% adapalene gel + antimicrobial agents (clindamycin gel or nadifloxacin cream) | 12 |
| Jones 2002 | USA | 4 | 18.5 | 0.498 | 223 | 1. 3% erythromycin + 5% benzoyl peroxide  2. vehicle control | 8 |
| Jung 2014 | Korea | 1 | 23.7 | 0.2 | 30 | 1. Omega-3 Fatty Acid  2. Gamma-linolenic Acid  3. no treatment | 10 |
| Kaminaka 2014 | Japan | 1 | 25 | 0.64 | 50 | 1. Glycolic Acid Chemical Peeling  2. Placebo | 10 |
| Karsai 2010 | Germany | 1 | 19.7 | 0.52 | 70 | 1. 1% clindamycin + 5% benzoyl peroxide + laser  2. 1% clindamycin + 5% benzoyl peroxide | 4 |
| Katsambas 1987 | Greece | 1 | NA | 0.95 | 60 | 1. clindamycin phosphate 1%  2. tetracyline hydrochloride 500mg | 12 |
| Kawashima 2014 | Japan, USA | 19 | 21.9 | 0.65 | 360 | 1. 3% benzoyl peroxide  2. vehicle | 12 |
| Kawashima 2015 | Japan, USA | 26 | 20.8 | 0.66 | 799 | 1. clindamycin + 3% benzoyl peroxide (QD)  2. clindamycin + 3% benzoyl peroxide (BID)  3. clindamycin | 12 |
| Khodaeiani 2013 | Iran | 1 | 23.57 | 0.65 | 80 | 1. 1% Clindamycin  2. 4% Nicotinamide | 8 |
| Kim 2017 | Korea | 1 | 24.75 | 0.67 | 28 | 1. Non-ablative fractional laser-assisted daylight photodynamic therapy with topical Methyl-aminolevulinate  2. Daylight photodynamic therapy | 8 |
| Kwon 2015 | Korea | 2 | 22.75 | 0.48 | 46 | 1. Daylight photodynamic therapy with 3-butenyl 5-aminolevulinate hydrochloride  2. vehicle | 12 |
| Langner 2007a | Poland, UK | 2 | 20.35 | 0.66 | 148 | 1. clindamycin phosphate (1%) plus benzoyl peroxide (5%)  2. erythromycin (4%) plus zinc acetate (1.2%) | 12 |
| Langner 2007b | Poland, UK | 2 | 21.6 | 0.58 | 130 | 1. clindamycin phosphate + benzoyl peroxide (Duac)  2. adapalene 0.1% (Differin) | 12 |
| Lekwuttikarn 2017 | Thailand | 1 | 18 | 0.43 | 60 | 1. Pulsed dye laser  2. no treatment | 8 |
| Leyden 2006 | USA | 5 | 22 | 0.55 | 137 | 1. 0.1% tazarotenegel  2. 100-mg minocycline capsule  3. 0.1% tazarotenegel + minocycline capsule | 12 |
| Lu 2016 | Taiwan | 1 | 29.1 | 1 | 64 | 1. Epigallocatechin-3-gallate (a decaffeinated green tea extract)  2. placebo | 4 |
| Lueangarun 2018 | Thailand | 1 | 25.14 | 0.85 | 50 | 1. 0.5% topical mangosteen extract + benzoyl peroxide  2. 1% clindamycin gel + benzoyl peroxide | 12 |
| Marazzi 2002 | UK | 11 | 17 | 0.56 | 188 | 1. 0.1% isotretinoin + 4% erythromycin  2. 5% benzoyl peroxide + 3% erythromycin | 12 |
| Moftah 2016 | Egypt | 3 | 23.7 | 0.4 | 70 | 1. Intense pulsed light  2. photodynamic therapy using liposomal methylene blue gel | 4 |
| Mohebbipour 2015 | Iran | 1 | 23.5 | 0.72 | 50 | 1. sunflower seeds  2. no treatment | 2 |
| Moneib 2014 | Egypt | 1 | 21.5 | 0.79 | 48 | 1. 1550-nm erbium glass laser  2. no treatment | 2 |
| Nestor 2016 | USA | 1 | 20.2 | 0.3 | 92 | 1. 445nm blue/630nm red light therapy mask  2. 2.5% benzoyl peroxide  3. 445nm blue/630nm red light therapy mask + topical 1% salicylic acid | 12 |
| Nicklas 2018 | Chile | 1 | 21 | 0.43 | 46 | 1. aminolaevulinic acid photodynamic therapy  2. adapalene gel + doxycycline | 12 |
| Nikolis 2017 | Canada, Italy, Greece | 5 | 21.6 | 0.71 | 49 | 1. an LED blue light device using photo-converter chromophores  2. no treatment | 12 |
| Orringer 2004 | USA | 1 | 20.7 | 0.4 | 38 | 1. pulsed dye laser  2. no treatment | 12 |
| Ozgen 2013 | Turkey | 1 | 19.37 | 0.61 | 93 | 1. 1% Nadifloxacin  2. 1% Nadifloxacin + Benzoyl peroxide | 8 |
| Papageorgiou 2000 | UK | 1 | 27.73 | 0.73 | 41 | 1. Nels cream (Chloroxylenol and zinc oxide)  2. 5% benzoyl peroxide  3. vehicle | 8 |
| Pariser 2016 | USA | 15 | 17 | 0.43 | 153 | 1. Photodynamic therapy with methyl aminolaevulinate  2. vehicle | 12 |
| Poláková 2015 | Slovakia, France | 13 | 18.7 | 0.7 | 111 | 1. a dermocosmetic (containing bakuchiol, Ginkgo biloba extract and mannitol) + adapalene 0.1% gel  2. vehicle cream + adapalene 0.1% gel | 8 |
| Poli 2005 | France | >1* | 19.15 | 0.31 | 81 | 1. combination of 0.1% retinaldehyde and 6% glycolic acid (Diacnéal)  2. vehicle | 13 |
| Poulin 2011 | Canada, Puerto Rico, USA, France | >1* | 18.6 | 0.457 | 243 | 1. doxycycline 100 mg daily and adapalene + benzoyl peroxide (Epiduo)  2. vehicle + doxycycline 100 mg daily | 24 |
| Richter 2015 | Germany | 1 | 20.7 | 0.62 | 48 | 1. Tyrothricin 0.1%  2. Clindamycin + benzoyl peroxide 5%  3. benzoyl peroxide 5% | 3.5 |
| Rizer 2001 | USA | >1* | 19.4 | 0.531 | 667 | 1. topical clindamycin formulation (Clindagel) –QD  2. vehicle -QD  3. topical clindamycin formulation (Clindagel) -BID  4. vehicle -BID  5. topical clindamycin formulation (Cleocin T) -BID | 12 |
| Schaller 2016 | Germany | 11 | 20.1 | 0.548 | 222 | 1. benzoyl peroxide 3% + clindamycin 1% (Duac)  2. Azelaic acid 20% (Skinoren) | 12 |
| Seaton 2003 | UK | 1 | 28.5 | 0.63 | 36 | 1. Pulsed-dye laser  2. sham laser | 12 |
| Tabasum 2014 | India | 1 | 21.15 | 0.575 | 40 | 1. Unani preparation (Zimade Muhasa)  2. 5% benzoyl peroxide gel | 6 |
| Takigawa 2013 | Japan | >1* | 22.7 | 0.665 | 188 | 1. adapalene 0.1% gel + nadifloxacin 1% cream  2. adapalene 0.1% gel | 12 |
| Tan 2010 | UK, Canada, Germany, France, USA, Poland, Hungary, Puerto Rico | 157 | 18.3 | 0.52 | 3855 | 1. adapalene 0.1% + benzoyl peroxide 2.5%  2. adapalene 0.1%  3. benzoyl peroxide 2.5%  4. vehicle | 12 |
| Thiboutot 2002 | USA | 6 | 19.9 | 0.514 | 327 | 1. 3% erythromycin/5% benzoyl peroxide (EBP Park)  2. vehicle Park  3. 3% erythromycin/5% benzoyl peroxide (EBP Jar)  4. vehicle Jar | 8 |
| Thiboutot 2005 | USA | 35 | 17.85 | 0.464 | 467 | 1. adapalene gel 0.1% + oral lymecycline  2. vehicle + oral lymecycline | 12 |
| Thiboutot 2006 | USA, Canada | 33 | 18.2 | 0.5 | 653 | 1. Adapalene gel 0.3%  2. Adapalene gel 0.1%  3. Vehicle gel | 12 |
| Thiboutot 2008 | USA | >1* | 19.3 | 0.524 | 2813 | 1. Clindamycin- benzoyl peroxide 2.5%  2. Clindamycin phosphate 1.2%  3. benzoyl peroxide 2.5%  3. Vehicle | 12 |
| Thiboutot 2016 | USA | >1* | 20.25 | 0.558 | 4340 | 1. dapsone gel, 7.5%  2. vehicle | 12 |
| Tirado-Sanchez 2013 | Mexico | 1 | 20 | 0.55 | 171 | 1. Adapalene 0.1%  2. Adapalene 0.3%  3. Tretinoin 0.05%  4. Placebo | 13 |
| Wiegell 2006 | Denmark | 1 | 23.5 | 0.613 | 31 | 1. methyl aminolaevulinate-based photodynamic therapy  2. no treatment | 12 |
| Xu 2016 | China | 24 | 23.35 | 0.753 | 1016 | 1. clindamycin 1% + benzoyl peroxide 5%  2. clindamycin 1% | 12 |
| Xu 2017 | China | 1 | 24 | 0.568 | 95 | 1. minocycline hydrochloride capsule 100mg/d + photodynamic therapy with aminolevulinic acid  2. minocycline hydrochloride capsule alone | 8 |
| Yin 2010 | China | 1 | 24.96 | 0.54 | 180 | 1. 5% aminolaevulinic acid–photodynamic therapy  2. 10% aminolaevulinic acid–photodynamic therapy  3. 15% aminolaevulinic acid–photodynamic therapy  4. 20% aminolaevulinic acid–photodynamic therapy  5. vehicle | 24 |
| Zhang 2004 | China | >1* | 21.9 | 0.68 | 300 | 1. adapalene gel 0.1% + clindamycin phosphate 1%  2. clindamycin phosphate 1% | 12 |

Reference:

(Katsambas et al., 1987; Habbema et al., 1989; Hughes et al., 1992; Bojar et al., 1994; Fonseca et al., 1995; Glass et al., 1999; Papageorgiou and Chu, 2000; Rizer et al., 2001; Jones et al., 2002; Marazzi et al., 2002; Thiboutot et al., 2002, 2005, 2006, 2008, 2016; Seaton et al., 2003; Orringer et al., 2004; Zhang et al., 2004; Poli et al., 2005; Leyden et al., 2006; Wiegell and Wulf, 2006; Charakida et al., 2007; Langner et al., 2007, 2008; Haedersdal et al., 2008; Bissonnette et al., 2009, 2017; Karsai et al., 2010; Yin et al., 2010; Dréno et al., 2011, 2014; Poulin et al., 2011; Tan et al., 2011; Afzali et al., 2012; Akarsu et al., 2012; Capitanio et al., 2012; Hayashi and Kawashima, 2012; Khodaeiani et al., 2013; Takigawa et al., 2013; Tirado‐Sánchez et al., 2013; ÖZGEN and Gürbüz, 2013; Hajheydari et al., 2014; Jung et al., 2014; Kaminaka et al., 2014; Kawashima et al., 2014, 2015; Moneib et al., 2014; Cook-Bolden, 2015; Mohebbipour et al., 2015; Poláková et al., 2015; Chularojanamontri et al., 2016; Gold et al., 2016, 2019; Kwon et al., 2016; Lu and Hsu, 2016; Moftah et al., 2016; Nestor et al., 2016; Pariser et al., 2016; Richter et al., 2016; Schaller et al., 2016; Tabasum et al., 2016; Xu et al., 2016, 2017; Alba et al., 2017; Appiah et al., 2017; Bouloc et al., 2017; Kim et al., 2017; Lekwuttikarn et al., 2017; Hayashi et al., 2018; Ito et al., 2018; Lueangarun et al., 2018; Nikolis et al., 2018; Nicklas et al., 2019)

# Table 2 Risk of bias

| Study ID | Random sequence generation | Allocation concealment | Double-blinded | Lost to follow-up | ITT or not | Multicenter or not | Had a protocol or not |
| --- | --- | --- | --- | --- | --- | --- | --- |
| Afzali 2010 | - | - | + | - | No | No | No |
| Akarsu 2011 | - | - | single-blind | 0 | No | No | Yes |
| Alba 2016 | - | - | single-blind | - | No | No | No |
| Appiah 2017 | Minitab statistical software random data sampling function | - | + | - | No | No | Yes |
| Bissonnette 2009 | - | + | single-blind | 8\8 (20%) | No | Yes | No |
| Bissonnette 2016 | a computer-based randomization program | + | + | 0 | Yes | Yes | Yes |
| Bojar 1994 | - | - | + | 7 (13.4%) | No | No | No |
| Bouloc 2017 | - | - | - | 1\0\0 (<5%) | No | Yes | No |
| Capitanio 2012 | - | + | + | 0 | No | No | No |
| Charakida 2007 | a computer-generated sequence | - | + | 0 | Yes | No | Yes |
| Chularojanamontri 2015 | nQuery program | - | + | 0 | Yes | No | Yes |
| Cook-Bolden 2015 | - | - | + | - | Yes | Yes | No |
| Dreno 2011 | a computed randomization list that generated treatment numbers in a block size of four | + | + | 3\7 (<5%) | Yes | Yes | Yes |
| Dreno 2014 | - | - | + | 147\139\51\26 (7.9%) | Yes | Yes | Yes |
| Fonseca 1995 | - | - | + | 25 (9.1%) | No | Yes | Yes |
| Glass 1999 | computer-generated randomisation schedule | - | + | 0 | Yes | No | No |
| Gold 2016 | a randomization list | + | + | 12\8\4 (<5%) | Yes | Yes | Yes |
| Gold 2019 | - | - | + | 27\18 (<5%) | Yes | Yes | Yes |
| Habbema 1989 | - | - | + | - | No | Yes | No |
| Hædersdal 2008 | patients drawing lots | + | - | 0 | No | No | Yes |
| Hajheydari 2013 | - | - | + | 0 | No | No | Yes |
| Hayashi 2011 | - | + | open-label | 0\2\0 (<5%) | No | Yes | Yes |
| Hayashi 2018 | validated internal software | - | investigator-blind | 0 | Yes | Yes | Yes |
| Hughes 1992 | - | - | + | - | No | No | Yes |
| Ito 2018 | - | - | open-label | - | No | Yes | Yes |
| Jones 2002 | - | - | + | - | Yes | Yes | Yes |
| Jung 2014 | computer-generated randomisation | + | + | 0 | No | No | No |
| Kaminaka 2014 | - | - | + | 1 (<5%) | No | No | Yes |
| Karsai 2010 | a computer-generated randomization schedule | - | single-blinded | 7 (5.2%) | No | No | Yes |
| Katsambas 1987 | - | - | + | - | No | No | No |
| Kawashima 2014 | a computer randomization system | - | + | 1\0 (<5%) | Yes | Yes | Yes |
| Kawashima 2015 | a computer randomisation system | + | investigator-blinded | 0 | Yes | Yes | Yes |
| Khodaeiani 2013 | - | - | + | - | No | No | Yes |
| Kim 2017 | computer-based random number generators | - | - | 1\1 (7.1%) | No | No | Yes |
| Kwon 2015 | computer-based random number generators | + | + | 1 (<5%) | Yes | Yes | Yes |
| Langner 2007a | a computer-generated randomization schedule with a block size of 6 | - | investigator-blinded | 2\6 (5.4%) | Yes | Yes | No |
| Langner 2007b | a computer-generated randomization schedule with a block size of 6 | - | investigator-blinded | 1\2 (<5%) | Yes | Yes | Yes |
| Lekwuttikarn 2017 | - | - | investigator-blinded | - | No | No | Yes |
| Leyden 2006 | computer-generated randomization schedule (using a block size of 6) | + | + | 4\2\2 (7.2%) | No | Yes | No |
| Lu 2016 | a computed randomization list | + | + | 6\5 (13.7%) | No | No | Yes |
| Lueangarun 2018 | - | - | + | 3 (10.7%) | Yes | No | Yes |
| Marazzi 2002 | a pre-determined randomization schedule | - | single-blinded | 3\0 (<5%) | Yes | Yes | Yes |
| Moftah 2016 | - | - | - | 0 | No | Yes | Yes |
| Mohebbipour 2015 | Microsoft Excel software package | + | - | 0 | No | No | Yes |
| Moneib 2014 | tossing a coin | - | + | - | No | No | No |
| Nestor 2016 | - | - | + | - | Yes | No | Yes |
| Nicklas 2018 | computer generated random numbers | + | investigator-blinded | 0 | Yes | No | Yes |
| Nikolis 2017 | - | - | open-label | 0 | Yes | Yes | Yes |
| Orringer 2004 | a table of random numbers | - | investigator-blinded | 3\3 (15%) | Yes | No | Yes |
| Ozgen 2013 | a computer-generated list | - | + | 3 (<5%) | Yes | No | Yes |
| Papageorgiou 2000 | - | - | + | 4 (8.8%) | No | No | No |
| Pariser 2016 | a block randomization list | + | + | 0\2 (<5%) | Yes | Yes | Yes |
| Poláková 2015 | - | - | + | - | No | Yes | No |
| Poli 2005 | - | - | + | 2 (<5%) | Yes | Yes | Yes |
| Poulin 2011 | - | + | + | 5\11 (6.5%) | Yes | Yes | Yes |
| Richter 2015 | a computer-generated list | + | observer-blind\investigator-blinded | 0 | No | No | Yes |
| Rizer 2001 | - | - | evaluator-blind | - | Yes | Yes | Yes |
| Schaller 2016 | a computer-generated schedule | - | assessor-blinded | 0\3 (<5%) | Yes | Yes | Yes |
| Seaton 2003 | a computer-generated sequence | + | + | 0 | Yes | No | Yes |
| Tabasum 2014 | a computer-generated randomization table | - | Investigator-Blind | 3\2 (12.5%) | No | No | Yes |
| Takigawa 2013 | - | + | Investigator-Blind | 2\1 (<5%) | Yes | Yes | Yes |
| Tan 2010 | - | - | + | 51\54\44\55 (5.2%) | Yes | Yes | Yes |
| Thiboutot 2002 | - | - | + | - | Yes | Yes | Yes |
| Thiboutot 2005 | - | - | Investigator-Blind | 26\14 (8.5%) | Yes | Yes | Yes |
| Thiboutot 2006 | - | + | + | 8\12\5 (<5%) | Yes | Yes | Yes |
| Thiboutot 2008 | permuted blocks within strata | - | + | 32\49\49\27 (5.5%) | Yes | Yes | Yes |
| Thiboutot 2016 | - | - | + | 83\69 (<5%) | Yes | Yes | Yes |
| Tirado-Sanchez 2013 | - | - | + | 1\1\2\0 (<5%) | No | No | No |
| Wiegell 2006 | - | - | investigator-blinded | 1\1 (5.5%) | No | No | Yes |
| Xu 2016 | a computer-generated randomization schedule | - | assessor-blind | 22\26 (9.4%) | Yes | Yes | Yes |
| Xu 2017 | computer-generated random numbers | + | - | - | Yes | No | Yes |
| Yin 2010 | SAS software | - | single-blinded | 0 | No | No | Yes |
| Zhang 2004 | - | - | investigator-blinded\open-labelled | 9 (<5%) | Yes | Yes | Yes |

# Table 3 Meta-regression of non-inflammatory lesions reduction

| Covariate | Median coefficient | 95%Cr | Between-study standard deviation | I-square | DIC |
| --- | --- | --- | --- | --- | --- |
| No regression | - | - | 3.238[1.98, 5.05] | 11% | 291 |
| **Duration of treatment** | **-3.9955** | **[-6.792, -0.5273]** | **2.399[1.19, 4.33]** | **15%** | **295** |
| Double-blinded | -3.5134 | [-8.227, 1.0866] | 3.06[1.78, 4.88] | 12% | 291.6 |
| Lost to follow-up  (<5% or not) | 0.22 | [-3.557, 4.573] | 3.36[2.04, 5.22] | 11% | 291.5 |
| Samplesize | -0.5053 | [-2.900, 2.003] | 3.3[1.995, 5.188] | 11% | 291.4 |
| Age | -0.7934 | [-5.265, 3.2261] | 3.36[2, 5.25] | 10% | 291 |
| Proportion of women | 1.238 | [-1.96, 4.023] | 3.1[1.76, 5.03] | 12% | 293.5 |
| **Low quality or not** | **-10.668** | **[-19.231, -2.2532]** | **2.99[1.823, 4.69]** | **10%** | **288** |
| ITT or not | -0.1492 | [-3.842, 3.729] | 3.32[2, 5.2] | 11% | 291.7 |
| Split-face or not | -2.0550 | [-6.865, 3.4013] | 3.22[1.93, 5.1] | 11% | 291.8 |

# Table 4 SUCRA of non-inflammatory lesions reduction after adjustment

| **SUCRA** | Original model | Covariate of treatment duration | Covariate of low quality or not | Uniform prior | Lognormal prior | Half-normal prior |
| --- | --- | --- | --- | --- | --- | --- |
| TR+BPO | 94% | 95% | 95% | 94% | 95% | 95% |
| TR+BPO+MTCAM | 94% | 94% | 94% | 94% | 95% | 95% |
| TA+BPO+CP | 91% | 91% | 91% | 91% | 91% | 92% |
| TA+TR+MTCAM | 79% | 80% | 81% | 80% | 81% | 82% |
| TA+TR | 75% | 75% | 76% | 75% | 76% | 77% |
| TA+BPO | 74% | 75% | 75% | 74% | 75% | 75% |
| TR+BPO+OA | 73% | 73% | 75% | 73% | 76% | 80% |
| BPO+MTCAM | 68% | 70% | 70% | 68% | 69% | 71% |
| TA+MTCAM | 66% | 67% | 68% | 66% | 67% | 68% |
| TA+BPO+Lasers | 63% | 64% | 65% | 63% | 65% | 63% |
| **BPO** | 59% | 59% | 58% | 58% | 56% | **48%** |
| **Lasers+PDT** | 56% | 59% | **36%** | 56% | 58% | 63% |
| TR+MTCAM | 56% | 54% | 59% | 56% | 57% | 60% |
| LED | 55% | 54% | 56% | 55% | 53% | 48% |
| TA | 54% | 54% | 57% | 54% | 56% | 59% |
| **MTCAM** | 45% | 51% | 45% | 45% | 41% | **30%** |
| CP | 40% | 41% | 43% | 40% | 39% | 39% |
| TR | 38% | 35% | 42% | 38% | 39% | 41% |
| **LED+MTCAM** | 35% | 35% | 37% | 35% | 31% | **25%** |
| **PDT** | 34% | **16%** | 37% | 34% | 35% | 41% |
| TR+OA | 33% | 30% | 38% | 33% | 35% | 39% |
| **IPL** | 30% | **14%** | 34% | 30% | 30% | 32% |
| **Lasers** | 29% | **44%** | **9%** | 29% | 29% | 32% |
| TD | 28% | 27% | 32% | 27% | 27% | 26% |
| OA+PDT | 26% | 24% | 30% | 26% | 26% | 28% |
| **TS** | 23% | **34%** | **3%** | 23% | 22% | 21% |
| TT | 18% | 18% | 21% | 17% | 15% | 11% |
| Placebo | 12% | 15% | 16% | 12% | 11% | 11% |
| OA | 2% | 2% | 6% | 2% | 2% | 2% |

# Table 5 Meta-regression of inflammatory lesions reduction

| Covariate | Median coefficient | 95%Cr | Between-study standard deviation | I-square | DIC |
| --- | --- | --- | --- | --- | --- |
| No regression | - | - | 3.8[2.9, 5.02] | 4% | 294 |
| Duration of treatment | 2.7886 | [-0.806, 6.4645] | 3.78[2.9, 5] | 3% | 293 |
| **Double-blinded** | **-9.653** | **[-13.2, -6.28]** | **2.6[1.86, 3.63]** | **7%** | **294.4** |
| Lost to follow-up  (<5% or not) | 2.05 | [-1.67, 5.97] | 3.84[2.92, 5.08] | 3% | 294 |
| Samplesize | -0.196 | [-2.6, 2.37] | 3.87[2.95, 5.14] | 4% | 294 |
| Age | -0.855 | [-4.55, 2.84] | 3.84[2.93, 5.08] | 4% | 294.6 |
| Proportion of women | -2.273 | [-5.166, 0.593] | 3.727[2.84, 4.93] | 4% | 294 |
| Low quality or not | -3.699 | [-9.89, 2.43] | 3.78[2.88, 5] | 4% | 294.4 |
| ITT or not | -0.97 | [-4.46, 2.79] | 3.85[2.94, 5.1] | 4% | 294 |
| **Split-face or not** | **5.79** | **[0.84, 11.1]** | **3.73[2.87, 4.92]** | **2%** | **291** |

# Table 6 SUCRA of inflammatory lesions reduction after adjustment

| **SUCRA** | Original model | Covariate of treatment duration | Covariate of low quality or not | Uniform prior | Lognormal prior | Half-normal prior |
| --- | --- | --- | --- | --- | --- | --- |
| TR+BPO | 81% | 81% | 81% | 81% | 82% | 85% |
| TR+BPO+MTCAM | 81% | 81% | 81% | 81% | 82% | 88% |
| LED | 77% | 76% | 76% | 77% | 78% | 79% |
| OA+PDT | 76% | 76% | 76% | 76% | 77% | 83% |
| TA+BPO+Lasers | 76% | 76% | 76% | 76% | 77% | 79% |
| BPO+MTCAM | 72% | 72% | 72% | 72% | 73% | 77% |
| TA+TR+MTCAM | 70% | 70% | 70% | 70% | 71% | 78% |
| TA+BPO | 68% | 68% | 68% | 68% | 68% | 70% |
| LED+MTCAM | 64% | 63% | 62% | 64% | 64% | 62% |
| Lasers+PDT | 63% | 62% | 62% | 63% | 62% | 57% |
| TR+BPO+OA | 63% | 63% | 63% | 63% | 64% | 70% |
| TA+BPO+CP | 61% | 62% | 62% | 62% | 62% | 66% |
| TA+MTCAM | 59% | 60% | 60% | 59% | 60% | 65% |
| BPO | 57% | 56% | 56% | 57% | 57% | 49% |
| TA+TR | 53% | 54% | 54% | 53% | 54% | 60% |
| **PDT** | 46% | 43% | 44% | 46% | 44% | **33%** |
| TR+MTCAM | 45% | 46% | 45% | 45% | 45% | 48% |
| TA | 43% | 44% | 44% | 43% | 43% | 47% |
| TS | 38% | 37% | 38% | 38% | 38% | 37% |
| **Lasers** | 37% | 38% | 37% | 37% | 35% | **24%** |
| MTCAM | 37% | 34% | 34% | 37% | 36% | 29% |
| TT | 37% | 37% | 36% | 37% | 36% | 30% |
| TR+OA | 33% | 33% | 33% | 33% | 33% | 34% |
| OA | 30% | 30% | 30% | 30% | 29% | 30% |
| TR | 27% | 28% | 28% | 27% | 27% | 30% |
| CP | 24% | 24% | 24% | 24% | 23% | 19% |
| TD | 18% | 21% | 20% | 18% | 17% | 12% |
| **IPL** | 11% | 11% | 11% | 11% | 10% | **3%** |
| Placebo | 5% | 5% | 5% | 5% | 5% | 4% |

# File 1 Protocol amendments

We deviated from the protocol by some changes which are as follows:

1. In order to make the quality of primary studies controllable enough to be peer-reviewed, we only searched in PubMed, Embase and CENTRAL and searching restriction was limited in English articles. And because of the limited study information in ClinicalTrials and ICTRP, we had to give up studies from there. Thus, Two Chinese databases (CNKI, WanFang database) and additional sources (ClinicalTrials, ICTRP) were excluded.

2. Because subjects of many trials covered teenagers and adults, we included participants of all ages except children.

3. Because many trials only reported lesions count or that changed from baseline, we changed primary outcomes from percent reduction into count reduction. And many trials did not report skin lesion scores or life quality scores or reported in different criteria, so we removed all types of score in additional outcomes.

4. We conducted some additional analyses, including frequentist network meta-analysis and pairwise meta-analysis, in order to have a robust result.

# File 2 Search strategy

PubMed:

#1 Search ((acne vulgaris[MeSH Terms]) OR acne vulgaris[Title/Abstract]) OR acne[Title/Abstract]

17286

#2 Search (((((((randomized controlled study[Title/Abstract]) OR randomized controlled trial[Title/Abstract]) OR randomized study[Title/Abstract]) OR randomized trial[Title/Abstract]) OR randomized parallel-group study[Title/Abstract]) OR double-blinded controlled study[Title/Abstract]) OR randomized vehicle-controlled study[Title/Abstract]) OR randomized placebo-controlled study[Title/Abstract]

127957

#3 Search ((Meta-analysis[Title/Abstract]) OR systematic review[Title/Abstract]) OR protocol[Title/Abstract]

480956

#4 Search #1 AND #2 NOT #3

158

Embase:

#1 ('acne'/exp OR acne) AND vulgaris OR 'acne'/exp OR acne

38670

#2 (((((((randomized AND controlled AND ('study'/exp OR study) OR randomized) AND controlled AND ('trial'/exp OR trial) OR randomized) AND ('study'/exp OR study) OR randomized) AND ('trial'/exp OR trial) OR randomized) AND 'parallel group' AND ('study'/exp OR study) OR 'double blinded') AND controlled AND ('study'/exp OR study) OR randomized) AND 'vehicle controlled' AND ('study'/exp OR study) OR randomized) AND 'placebo controlled' AND ('study'/exp OR study)

82102

#3 ('meta analysis'/exp OR 'meta analysis' OR systematic) AND ('review'/exp OR review) OR 'protocol'/exp OR protocol

837358

#4 #1 AND #2 NOT #3

349

CENTRAL:

#1 (acne vulgaris):ti,ab,kw OR (acne):ti,ab,kw (Word variations have been searched)

4179

#2 (randomized controlled study):ti,ab,kw OR (randomized controlled trial):ti,ab,kw OR (randomized study):ti,ab,kw OR (randomized trial):ti,ab,kw OR (randomized parallel-group study):ti,ab,kw (Word variations have been searched)

845758

#3 (protocol):ti,ab,kw (Word variations have been searched)

99961

#4 (#1 AND #2) NOT #3

2291

# File 3 Complete statistical analysis

A Bayesian method was used to perform pairwise meta-analyses and network meta-analyses. The pairwise analysis was pooled when at least two trials assessed the same intervention and comparator for the same outcome. And when they formed a connected net of treatments, the network meta-analysis (NMA) was performed to synthesize networks of trials comparing each treatment at the same time. To account for the between-study heterogeneity and to attain greater generalizability for pooled results, all the analyses were carried out under a random effect model. Moreover, the model was conducted with a vague prior adjusting for the correlation of multi-arm trials and assuming a common heterogeneity parameter (σ ~ Unif (0, N), N were set as 33.5 for the non-inflammatory data, 27.85 for the inflammatory data and 3 for the adverse event data, which were determined by the outcome measure scale). The network model was performed under consistency assumption, and a node-splitting analysis was used to examine this assumption with presented pooled direct and indirect estimates and inconsistency p-values for each split comparison(van Valkenhoef et al., 2016).

The primary outcomes, namely the mean change in non-inflammatory and inflammatory lesions count from baseline, were calculated as mean difference (MD), and the secondary outcome, response rate of adverse events, was calculated as (log) risk ratio (RR). The analyses of MD were performed under the model assumption of a normal likelihood and identity link function while the analysis of RR was performed under the model assumption of a binomial likelihood and ‘log’ link function.

We optimized the model and generated posterior samples using Markov-Chain Monte-Carlo methods running in four chains. We set at least 20000 adaptation iterations to get convergence and 100000 simulation iterations with a thinning factor of 10 to produce the outputs. We used the Brooks-Gelman-Rubin method to assess convergence of the model and calculated the ‘potential scale reduction factor’ for each comparison together with the confidence interval(Gelman and Rubin, 1992; Brooks and Gelman, 1998). Approximate convergence is diagnosed when the upper limit was close to 1.

We presented the network estimates (pooled of direct and indirect data) of each intervention compared with placebo and each other in forest plot and league table. The median MD and RR of posterior estimates were reported with their 95% credible intervals (95%CrI). We also ranked interventions by their posterior probability by calculating the Surface Under the Cumulative Ranking (SUCRA) curve values(Salanti et al., 2011). And we presented their median ranks along with the 95%CrIs. To show the simultaneous assessment of heterogeneity, we conducted an analysis of heterogeneity for the network with both direct and indirect results and quantitated it as I-square, which was calculated from Cochran’s Q value(Higgins et al., 2003).

To lower the impact of potential effect modifiers in the network from a continuous covariate or a subgroup effect or the baseline risk, we conducted the Bayesian meta-regression analysis to examine the robustness of effect estimates of primary outcomes with nine covariates(Dias et al., 2013). The following covariates were included: duration of treatment, double-blinded or not, lost to follow-up (<5% or not), the sample size of the trials, patients' age, the proportion of women, whether it is of low quality or not, whether it conducted ITT analysis or not, whether it is the split-face study or not. And then, we presented the posterior median of the interaction parameters (the covariates) and the corresponding 95% credible intervals and the deviance information criterion (DIC) which provides a measure of model fit that penalizes model complexity. The significant coefficients among covariates were adjusted, and corresponding SUCRA values were displayed afterward.

We also performed a network meta-analysis using frequentist methods with random effects based on the graph-theoretical method(Rücker, 2012). Afterward, we conducted an additive component network meta-analysis for combinations of treatments under the assumption that the effect of treatment combinations is the sum of the effects of its components, which means that common components cancel out in comparisons(Rücker et al., 2019). The rank of treatments was calculated by P-scores(Rücker and Schwarzer, 2015). Because two primary outcomes provided different ordering of treatments, we generated a biplot of P-scores with an overlay describing partial order of treatment ranks(Carlsen and Bruggemann, 2014). To find potential publication bias and small-study effects, we performed the comparison-adjusted funnel plots with the specified order by P-scores(Chaimani and Salanti, 2012).

In addition, we performed a pairwise meta-analysis using a random effect method with a restricted maximum-likelihood estimator for comparison of Miscellaneous therapies or Complementary and alternative medicine versus placebo. Because of the potential heterogeneity, we conducted a leave-one-out diagnostics for the pairwise model to examine the robustness.

All computations were done using R (V. 3.5.2) package gemtc(Valkenhoef, 2018) (V. 0.8-2) along with the Markov Chain Monte Carlo engine JAGS (V. 3.4.0) and package netmeta(Schwarzer, 2019) (V. 1.0-1) and package metafor(Viechtbauer, 2019) (V. 2.0-0). We performed the risk of bias graph using the Cochrane tool RevMan (V. 5.3).

# File 4 Choice of Prior Distributions

In Bayesian approaches, the prior distributions are very sensitive for the final results, and there is no reason to choose an informative prior distribution without an argument. Thus, we chose to set a non-informative prior distribution for the between-trial variance. That is, we place a uniform prior distribution on the standard deviation, and the upper limit of N represents a huge range of trial-specific treatment effect. If the median treatment effect was an MD of 10 and we set a prior of unif(0, N=10), then the expected 95% of MDs were between 0 and 20, i.e., 10±10.

Besides, we also added another three prior distributions in the sensitivity analysis. First, we set an alternative vague prior (σ ~ Unif (0, 5)). It represents a narrower interval of treatment effect compared with the primary analysis. Second, we set a weakly informative prior (τ ~ HN (1)). It represents a constraint to be positive and is the same as the meaning of normal distribution. Last, we set an informative prior distribution for a subjective outcome (τ ~ Lognormal (-2.01, 0.372)). The parameters of the distribution were taken from published meta-analyses recommended by Rhodes et al. (2015). This method of using external data was suggested by Higgins and Whitehead (1996). Prior distributions derived from large numbers of meta-analyses can be used with the same outcome and the similar type of interventions (Turner et al., 2012).

# File 5 Reference

Afzali, B. M., Yaghoobi, E., Yaghoobi, R., Bagherani, N., and Dabbagh, M. A. (2012). Comparison of the efficacy of 5% topical spironolactone gel and placebo in the treatment of mild and moderate acne vulgaris: a randomized controlled trial. *J Dermatolog Treat* 23, 21–25. doi:10.3109/09546634.2010.488260.

Akarsu, S., Fetil, E., Yücel, F., Gül, E., and Güneş, A. T. (2012). Efficacy of the addition of salicylic acid to clindamycin and benzoyl peroxide combination for acne vulgaris. *J. Dermatol.* 39, 433–438. doi:10.1111/j.1346-8138.2011.01405.x.

Alba, M. N., Gerenutti, M., Yoshida, V. M. H., and Grotto, D. (2017). Clinical comparison of salicylic acid peel and LED-Laser phototherapy for the treatment of Acne vulgaris in teenagers. *J Cosmet Laser Ther* 19, 49–53. doi:10.1080/14764172.2016.1247961.

Appiah, S., Lawley, B., Vu, M., Bell, C., and Jones, H. (2017). Evaluation of the effectiveness of Eladi Keram for the treatment of Acne vulgaris: a randomised controlled pilot study. *European Journal of Integrative Medicine* 12, 38–43. doi:10.1016/j.eujim.2017.04.004.

Bissonnette, R., Bolduc, C., Seité, S., Nigen, S., Provost, N., Maari, C., et al. (2009). Randomized study comparing the efficacy and tolerance of a lipophillic hydroxy acid derivative of salicylic acid and 5% benzoyl peroxide in the treatment of facial acne vulgaris. *J Cosmet Dermatol* 8, 19–23. doi:10.1111/j.1473-2165.2009.00418.x.

Bissonnette, R., Poulin, Y., Drew, J., Hofland, H., and Tan, J. (2017). Olumacostat glasaretil, a novel topical sebum inhibitor, in the treatment of acne vulgaris: A phase IIa, multicenter, randomized, vehicle-controlled study. *J. Am. Acad. Dermatol.* 76, 33–39. doi:10.1016/j.jaad.2016.08.053.

Bojar, R. A., Eady, E. A., Jones, C. E., Cunliffe, W. J., and Holland, K. T. (1994). Inhibition of erythromycin-resistant propionibacteria on the skin of acne patients by topical erythromycin with and without zinc. *Br. J. Dermatol.* 130, 329–336.

Bouloc, A., Roo, E., Imko-Walczuk, B., Moga, A., Chadoutaud, B., and Dréno, B. (2017). A skincare combined with combination of adapalene and benzoyl peroxide provides a significant adjunctive efficacy and local tolerance benefit in adult women with mild acne. *J Eur Acad Dermatol Venereol* 31, 1727–1731. doi:10.1111/jdv.14379.

Brooks, S. P., and Gelman, A. (1998). General Methods for Monitoring Convergence of Iterative Simulations. *Journal of Computational and Graphical Statistics* 7, 434–455. doi:10.1080/10618600.1998.10474787.

Capitanio, B., Sinagra, J. L., Weller, R. B., Brown, C., and Berardesca, E. (2012). Randomized controlled study of a cosmetic treatment for mild acne. *Clin. Exp. Dermatol.* 37, 346–349. doi:10.1111/j.1365-2230.2011.04317.x.

Carlsen, L., and Bruggemann, R. (2014). Partial order methodology: a valuable tool in chemometrics. *Journal of Chemometrics* 28, 226–234. doi:10.1002/cem.2569.

Chaimani, A., and Salanti, G. (2012). Using network meta-analysis to evaluate the existence of small-study effects in a network of interventions. *Research Synthesis Methods* 3, 161–176. doi:10.1002/jrsm.57.

Charakida, A., Charakida, M., and Chu, A. C. (2007). Double-blind, randomized, placebo-controlled study of a lotion containing triethyl citrate and ethyl linoleate in the treatment of acne vulgaris. *Br. J. Dermatol.* 157, 569–574. doi:10.1111/j.1365-2133.2007.08083.x.

Chularojanamontri, L., Tuchinda, P., Kulthanan, K., Varothai, S., and Winayanuwattikun, W. (2016). A double-blinded, randomized, vehicle-controlled study to access skin tolerability and efficacy of an anti-inflammatory moisturizer in treatment of acne with 0.1% adapalene gel. *Journal of Dermatological Treatment* 27, 140–145. doi:10.3109/09546634.2015.1079298.

Cook-Bolden, F. E. (2015). Efficacy and tolerability of a fixed combination of clindamycin phosphate (1.2%) and benzoyl peroxide (3.75%) aqueous gel in moderate or severe adolescent acne vulgaris. *J Clin Aesthet Dermatol* 8, 28–32.

Dias, S., Sutton, A. J., Welton, N. J., and Ades, A. E. (2013). Evidence Synthesis for Decision Making 3: Heterogeneity—Subgroups, Meta-Regression, Bias, and Bias-Adjustment. *Medical Decision Making* 33, 618–640. doi:10.1177/0272989X13485157.

Dréno, B., Bettoli, V., Ochsendorf, F., Layton, A. M., Perez, M., Dakovic, R., et al. (2014). Efficacy and safety of clindamycin phosphate 1.2%/tretinoin 0.025% formulation for the treatment of acne vulgaris: pooled analysis of data from three randomised, double-blind, parallel-group, phase III studies. *Eur J Dermatol* 24, 201–209. doi:10.1684/ejd.2014.2293.

Dréno, B., Kaufmann, R., Talarico, S., Torres Lozada, V., Rodríguez-Castellanos, M. A., Gómez-Flores, M., et al. (2011). Combination therapy with adapalene-benzoyl peroxide and oral lymecycline in the treatment of moderate to severe acne vulgaris: a multicentre, randomized, double-blind controlled study. *Br. J. Dermatol.* 165, 383–390. doi:10.1111/j.1365-2133.2011.10374.x.

Fonseca, E., Ferrándiz, C., Camarasa, J. G., Olmos, L., del Pino, J., Rodriguez, T., et al. (1995). Erythromycin lauryl sulphate in combination with tretinoin in the topical treatment of acne vulgaris. A multicentre double-blind clinical trial. *Journal of Dermatological Treatment* 6, 47–50. doi:10.3109/09546639509080591.

Gelman, A., and Rubin, D. B. (1992). Inference from Iterative Simulation Using Multiple Sequences. *Statist. Sci.* 7, 457–472. doi:10.1214/ss/1177011136.

Glass, D., Boorman, G. C., Stables, G. I., Cunliffe, W. J., and Goode, K. (1999). A placebo-controlled clinical trial to compare a gel containing a combination of isotretinoin (0.05%) and erythromycin (2%) with gels containing isotretinoin (0.05%) or erythromycin (2%) alone in the topical treatment of acne vulgaris. *Dermatology (Basel)* 199, 242–247. doi:10.1159/000018255.

Gold, L. S., Dhawan, S., Weiss, J., Draelos, Z. D., Ellman, H., and Stuart, I. A. (2019). A novel topical minocycline foam for the treatment of moderate-to-severe acne vulgaris: Results of 2 randomized, double-blind, phase 3 studies. *J. Am. Acad. Dermatol.* 80, 168–177. doi:10.1016/j.jaad.2018.08.020.

Gold, L. S., Weiss, J., Rueda, M. J., Liu, H., and Tanghetti, E. (2016). Moderate and Severe Inflammatory Acne Vulgaris Effectively Treated with Single-Agent Therapy by a New Fixed-Dose Combination Adapalene 0.3 %/Benzoyl Peroxide 2.5 % Gel: A Randomized, Double-Blind, Parallel-Group, Controlled Study. *Am J Clin Dermatol* 17, 293–303. doi:10.1007/s40257-016-0178-4.

Habbema, L., Koopmans, B., Menke, H. E., Doornweerd, S., and Boulle, K. D. (1989). A 4% erythromycin and zinc combination (Zineryt®) versus 2% erythromycin (Eryderm®) in acne vulgaris: a randomized, double-blind comparative study. *British Journal of Dermatology* 121, 497–502. doi:10.1111/j.1365-2133.1989.tb15518.x.

Haedersdal, M., Togsverd-Bo, K., Wiegell, S. R., and Wulf, H. C. (2008). Long-pulsed dye laser versus long-pulsed dye laser-assisted photodynamic therapy for acne vulgaris: A randomized controlled trial. *J. Am. Acad. Dermatol.* 58, 387–394. doi:10.1016/j.jaad.2007.11.027.

Hajheydari, Z., Saeedi, M., Morteza-Semnani, K., and Soltani, A. (2014). Effect of Aloe vera topical gel combined with tretinoin in treatment of mild and moderate acne vulgaris: a randomized, double-blind, prospective trial. *J Dermatolog Treat* 25, 123–129. doi:10.3109/09546634.2013.768328.

Hayashi, N., and Kawashima, M. (2012). Multicenter randomized controlled trial on combination therapy with 0.1% adapalene gel and oral antibiotics for acne vulgaris: Comparison of the efficacy of adapalene gel alone and in combination with oral faropenem. *The Journal of Dermatology* 39, 511–515. doi:10.1111/j.1346-8138.2011.01450.x.

Hayashi, N., Kurokawa, I., Siakpere, O., Endo, A., Hatanaka, T., Yamada, M., et al. (2018). Clindamycin phosphate 1.2%/benzoyl peroxide 3% fixed-dose combination gel versus topical combination therapy of adapalene 0.1% gel and clindamycin phosphate 1.2% gel in the treatment of acne vulgaris in Japanese patients: A multicenter, randomized, investigator-blind, parallel-group study. *J. Dermatol.* 45, 951–962. doi:10.1111/1346-8138.14497.

Higgins, J. P. T., Thompson, S. G., Deeks, J. J., and Altman, D. G. (2003). Measuring inconsistency in meta-analyses. *BMJ* 327, 557–560. doi:10.1136/bmj.327.7414.557.

Hughes, B. R., Norris, J. F., and Cunliffe, W. J. (1992). A double-blind evaluation of topical isotretinoin 0.05%, benzoyl peroxide gel 5% and placebo in patients with acne. *Clin. Exp. Dermatol.* 17, 165–168.

Ito, K., Masaki, S., Hamada, M., Tokunaga, T., Kokuba, H., Tashiro, K., et al. (2018). Efficacy and Safety of the Traditional Japanese Medicine Keigairengyoto in the Treatment of Acne Vulgaris. *Dermatol Res Pract* 2018, 4127303. doi:10.1155/2018/4127303.

Jones, T., Mark, L., Monroe, E., Weiss, J., and Levy, S. (2002). A Multicentre, Double-Blind, Parallel-Group Study to Evaluate 3% Erythromycin/5% Benzoyl Peroxide Dual-Pouch Pack for Acne Vulgaris. *Clin. Drug Investig.* 22, 455–462. doi:10.2165/00044011-200222070-00006.

Jung, J., Kwon, H., Hong, J., Yoon, J., Park, M., Jang, M., et al. (2014). Effect of Dietary Supplementation with Omega-3 Fatty Acid and Gamma-linolenic Acid on Acne Vulgaris: A Randomised, Double-blind, Controlled Trial. *Acta Dermato Venereologica* 94, 521–525. doi:10.2340/00015555-1802.

Kaminaka, C., Uede, M., Matsunaka, H., Furukawa, F., and Yamomoto, Y. (2014). Clinical evaluation of glycolic acid chemical peeling in patients with acne vulgaris: a randomized, double-blind, placebo-controlled, split-face comparative study. *Dermatol Surg* 40, 314–322. doi:10.1111/dsu.12417.

Karsai, S., Schmitt, L., and Raulin, C. (2010). The pulsed-dye laser as an adjuvant treatment modality in acne vulgaris: a randomized controlled single-blinded trial. *Br. J. Dermatol.* 163, 395–401. doi:10.1111/j.1365-2133.2010.09806.x.

Katsambas, A., Towarky, A. A., and Stratigos, J. (1987). Topical clindamycin phosphate compared with oral tetracycline in the treatment of acne vulgaris. *Br. J. Dermatol.* 116, 387–391.

Kawashima, M., Hashimoto, H., Alio Sáenz, A. B., Ono, M., and Yamada, M. (2014). Is benzoyl peroxide 3% topical gel effective and safe in the treatment of acne vulgaris in Japanese patients? A multicenter, randomized, double-blind, vehicle-controlled, parallel-group study. *J. Dermatol.* 41, 795–801. doi:10.1111/1346-8138.12580.

Kawashima, M., Hashimoto, H., Alió Sáenz, A. B., Ono, M., and Yamada, M. (2015). Clindamycin phosphate 1·2%-benzoyl peroxide 3·0% fixed-dose combination gel has an effective and acceptable safety and tolerability profile for the treatment of acne vulgaris in Japanese patients: a phase III, multicentre, randomised, single-blinded, acti. *British Journal of Dermatology* 172, 494–503. doi:10.1111/bjd.13265.

Khodaeiani, E., Fouladi, R. F., Amirnia, M., Saeidi, M., and Karimi, E. R. (2013). Topical 4% nicotinamide vs. 1% clindamycin in moderate inflammatory acne vulgaris. *Int. J. Dermatol.* 52, 999–1004. doi:10.1111/ijd.12002.

Kim, T. I., Ahn, H.-J., Kang, I. H., Jeong, K.-H., Kim, N. I., and Shin, M. K. (2017). Nonablative fractional laser-assisted daylight photodynamic therapy with topical methyl aminolevulinate for moderate to severe facial acne vulgaris: Results of a randomized and comparative study. *Photodermatology, Photoimmunology & Photomedicine* 33, 253–259. doi:10.1111/phpp.12312.

Kwon, H. H., Moon, K. R., Park, S. Y., Yoon, J. Y., Suh, D. H., and Lee, J. B. (2016). Daylight photodynamic therapy with 1.5% 3-butenyl 5-aminolevulinate gel as a convenient, effective and safe therapy in acne treatment: A double-blind randomized controlled trial. *J. Dermatol.* 43, 515–521. doi:10.1111/1346-8138.13191.

Langner, A., Chu, A., Goulden, V., and Ambroziak, M. (2008). A randomized, single-blind comparison of topical clindamycin + benzoyl peroxide and adapalene in the treatment of mild to moderate facial acne vulgaris. *British Journal of Dermatology* 158, 122–129. doi:10.1111/j.1365-2133.2007.08308.x.

Langner, A., Sheehan‐Dare, R., and Layton, A. (2007). A randomized, single-blind comparison of topical clindamycin + benzoyl peroxide (Duac®) and erythromycin + zinc acetate (Zineryt®) in the treatment of mild to moderate facial acne vulgaris. *Journal of the European Academy of Dermatology and Venereology* 21, 311–319. doi:10.1111/j.1468-3083.2006.01884.x.

Lekwuttikarn, R., Tempark, T., Chatproedprai, S., and Wananukul, S. (2017). Randomized, controlled trial split-faced study of 595-nm pulsed dye laser in the treatment of acne vulgaris and acne erythema in adolescents and early adulthood. *International Journal of Dermatology* 56, 884–888. doi:10.1111/ijd.13631.

Leyden, J., Thiboutot, D. M., Shalita, A. R., Webster, G., Washenik, K., Strober, B. E., et al. (2006). Comparison of Tazarotene and Minocycline Maintenance Therapies in Acne Vulgaris: A Multicenter, Double-blind, Randomized, Parallel-Group Study. *Arch Dermatol* 142, 605–612. doi:10.1001/archderm.142.5.605.

Lu, P. H., and Hsu, C. H. (2016). Does supplementation with green tea extract improve acne in post-adolescent women? A randomized, double-blind, and placebo-controlled clinical trial. *Complement Ther Med* 25, 159–163. doi:10.1016/j.ctim.2016.03.004.

Lueangarun, S., Sriviriyakul, K., Tempark, T., Managit, C., and Sithisarn, P. (2018). Clinical efficacy of 0.5% topical mangosteen extract in nanoparticle loaded gel in treatment of mild-to-moderate acne vulgaris: A 12-week, split-face, double-blinded, randomized, controlled trial. *Journal of Cosmetic Dermatology* 0. doi:10.1111/jocd.12856.

Marazzi, P., Boorman, G. C., Donald, A. E., and Davies, H. D. (2002). Clinical evaluation of Double Strength Isotrexin versus Benzamycin in the topical treatment of mild to moderate acne vulgaris. *J Dermatolog Treat* 13, 111–117. doi:10.1080/09546630260199460.

Moftah, N. H., Ibrahim, S. M., and Wahba, N. H. (2016). Intense pulsed light versus photodynamic therapy using liposomal methylene blue gel for the treatment of truncal acne vulgaris: a comparative randomized split body study. *Arch. Dermatol. Res.* 308, 263–268. doi:10.1007/s00403-016-1639-6.

Mohebbipour, A., Sadeghi-Bazargani, H., and Mansouri, M. (2015). Sunflower Seed and Acne Vulgaris. *Iran Red Crescent Med J* 17, e16544. doi:10.5812/ircmj.16544.

Moneib, H., Tawfik, A. A., Youssef, S. S., and Fawzy, M. M. (2014). Randomized split-face controlled study to evaluate 1550-nm fractionated erbium glass laser for treatment of acne vulgaris--an image analysis evaluation. *Dermatol Surg* 40, 1191–1200. doi:10.1097/DSS.0000000000000167.

Nestor, M. S., Swenson, N., Macri, A., Manway, M., and Paparone, P. (2016). Efficacy and Tolerability of a Combined 445nm and 630nm Over-the-counter Light Therapy Mask with and without Topical Salicylic Acid versus Topical Benzoyl Peroxide for the Treatment of Mild-to-moderate Acne Vulgaris. *J Clin Aesthet Dermatol* 9, 25–35.

Nicklas, C., Rubio, R., Cárdenas, C., and Hasson, A. (2019). Comparison of efficacy of aminolaevulinic acid photodynamic therapy vs. adapalene gel plus oral doxycycline for treatment of moderate acne vulgaris–A simple, blind, randomized, and controlled trial. *Photodermatology, Photoimmunology & Photomedicine* 35, 3–10. doi:10.1111/phpp.12413.

Nikolis, A., Fauverghe, S., Scapagnini, G., Sotiriadis, D., Kontochristopoulos, G., Petridis, A., et al. (2018). An extension of a multicenter, randomized, split-face clinical trial evaluating the efficacy and safety of chromophore gel-assisted blue light phototherapy for the treatment of acne. *Int. J. Dermatol.* 57, 94–103. doi:10.1111/ijd.13814.

Orringer, J. S., Kang, S., Hamilton, T., Schumacher, W., Cho, S., Hammerberg, C., et al. (2004). Treatment of acne vulgaris with a pulsed dye laser: a randomized controlled trial. *JAMA* 291, 2834–2839. doi:10.1001/jama.291.23.2834.

ÖZGEN, Z. Y., and Gürbüz, O. (2013). A randomized, double-blind comparison of nadifloxacin 1% cream alone and with benzoyl peroxide 5% lotion in the treatment of mild to moderate facial acne vulgaris. *Marmara Medical Journal* 26, 17–20. doi:10.5472/MMJ/2012.02649.

Papageorgiou, P. P., and Chu, A. C. (2000). Chloroxylenol and zinc oxide containing cream (Nels cream) vs. 5% benzoyl peroxide cream in the treatment of acne vulgaris. A double-blind, randomized, controlled trial. *Clin. Exp. Dermatol.* 25, 16–20.

Pariser, D. M., Eichenfield, L. F., Bukhalo, M., Waterman, G., Jarratt, M., and the PDT Study Group (2016). Photodynamic therapy with methyl aminolaevulinate 80 mg g ^−1^ for severe facial acne vulgaris: a randomized vehicle-controlled study. *British Journal of Dermatology* 174, 770–777. doi:10.1111/bjd.14345.

Poláková, K., Fauger, A., Sayag, M., and Jourdan, E. (2015). A dermocosmetic containing bakuchiol, Ginkgo biloba extract and mannitol improves the efficacy of adapalene in patients with acne vulgaris: result from a controlled randomized trial. *Clin Cosmet Investig Dermatol* 8, 187–191. doi:10.2147/CCID.S81691.

Poli, F., Ribet, V., Lauze, C., Adhoute, H., and Morinet, P. (2005). Efficacy and safety of 0.1% retinaldehyde/ 6% glycolic acid (diacneal) for mild to moderate acne vulgaris. A multicentre, double-blind, randomized, vehicle-controlled trial. *Dermatology (Basel)* 210 Suppl 1, 14–21. doi:10.1159/000081498.

Poulin, Y., Sanchez, N. P., Bucko, A., Fowler, J., Jarratt, M., Kempers, S., et al. (2011). A 6-month maintenance therapy with adapalene-benzoyl peroxide gel prevents relapse and continuously improves efficacy among patients with severe acne vulgaris: results of a randomized controlled trial: Adapalene-BPO maintenance therapy prevents relapse. *British Journal of Dermatology* 164, 1376–1382. doi:10.1111/j.1365-2133.2011.10344.x.

Richter, C., Trojahn, C., Hillmann, K., Dobos, G., Stroux, A., Kottner, J., et al. (2016). Reduction of Inflammatory and Noninflammatory Lesions with Topical Tyrothricin 0.1% in the Treatment of Mild to Severe Acne Papulopustulosa: A Randomized Controlled Clinical Trial. *Skin Pharmacol Physiol* 29, 1–8. doi:10.1159/000439439.

Rizer, R. L., Sklar, J. L., Whiting, D., Bucko, A., Shavin, J., and Jarratt, M. (2001). Clindamycin phosphate 1% gel in acne vulgaris. *Adv Ther* 18, 244–252.

Rücker, G. (2012). Network meta-analysis, electrical networks and graph theory. *Research Synthesis Methods* 3, 312–324. doi:10.1002/jrsm.1058.

Rücker, G., Petropoulou, M., and Schwarzer, G. (2019). Network meta-analysis of multicomponent interventions. *Biometrical Journal*. doi:10.1002/bimj.201800167.

Rücker, G., and Schwarzer, G. (2015). Ranking treatments in frequentist network meta-analysis works without resampling methods. *BMC Med Res Methodol* 15, 58. doi:10.1186/s12874-015-0060-8.

Salanti, G., Ades, A. E., and Ioannidis, J. P. A. (2011). Graphical methods and numerical summaries for presenting results from multiple-treatment meta-analysis: an overview and tutorial. *Journal of Clinical Epidemiology* 64, 163–171. doi:10.1016/j.jclinepi.2010.03.016.

Schaller, M., Sebastian, M., Ress, C., Seidel, D., and Hennig, M. (2016). A multicentre, randomized, single-blind, parallel-group study comparing the efficacy and tolerability of benzoyl peroxide 3%/clindamycin 1% with azelaic acid 20% in the topical treatment of mild-to-moderate acne vulgaris. *J Eur Acad Dermatol Venereol* 30, 966–973. doi:10.1111/jdv.13541.

Schwarzer, G. (2019). *Official Git repository of R package netmeta. Contribute to guido-s/netmeta development by creating an account on GitHub*. Available at: https://github.com/guido-s/netmeta [Accessed April 12, 2019].

Seaton, E. D., Charakida, A., Mouser, P. E., Grace, I., Clement, R. M., and Chu, A. C. (2003). Pulsed-dye laser treatment for inflammatory acne vulgaris: randomised controlled trial. *Lancet* 362, 1347–1352.

Tabasum, H., Ahmad, T., Anjum, F., and Rehman, H. (2016). The effect of Unani antiacne formulation (Zimade Muhasa) on acne vulgaris: A single-blind, randomized, controlled clinical trial. *Journal of Pakistan Association of Dermatology* 24, 319–326.

Takigawa, M., Tokura, Y., Shimada, S., Furukawa, F., Noguchi, N., Ito, T., et al. (2013). Clinical and bacteriological evaluation of adapalene 0.1% gel plus nadifloxacin 1% cream versus adapalene 0.1% gel in patients with acne vulgaris. *J. Dermatol.* 40, 620–625. doi:10.1111/1346-8138.12189.

Tan, J., Gollnick, H. P. M., Loesche, C., Ma, Y. M., and Gold, L. S. (2011). Synergistic efficacy of adapalene 0.1%-benzoyl peroxide 2.5% in the treatment of 3855 acne vulgaris patients. *J Dermatolog Treat* 22, 197–205. doi:10.3109/09546631003681094.

Thiboutot, D., Jarratt, M., Rich, P., Rist, T., Rodriguez, D., and Levy, S. (2002). A randomized, parallel, vehicle-controlled comparison of two erythromycin/benzoyl peroxide preparations for acne vulgaris. *Clin Ther* 24, 773–785.

Thiboutot, D. M., Kircik, L., McMichael, A., Cook-Bolden, F. E., Tyring, S. K., Berk, D. R., et al. (2016). Efficacy, Safety, and Dermal Tolerability of Dapsone Gel, 7.5% in Patients with Moderate Acne Vulgaris: A Pooled Analysis of Two Phase 3 Trials. *J Clin Aesthet Dermatol* 9, 18–27.

Thiboutot, D. M., Shalita, A. R., Yamauchi, P. S., Dawson, C., Arsonnaud, S., Kang, S., et al. (2005). Combination therapy with adapalene gel 0.1% and doxycycline for severe acne vulgaris: a multicenter, investigator-blind, randomized, controlled study. *Skinmed* 4, 138–146.

Thiboutot, D., Pariser, D. M., Egan, N., Flores, J., Herndon, J. H., Kanof, N. B., et al. (2006). Adapalene gel 0.3% for the treatment of acne vulgaris: a multicenter, randomized, double-blind, controlled, phase III trial. *J. Am. Acad. Dermatol.* 54, 242–250. doi:10.1016/j.jaad.2004.10.879.

Thiboutot, D., Zaenglein, A., Weiss, J., Webster, G., Calvarese, B., and Chen, D. (2008). An aqueous gel fixed combination of clindamycin phosphate 1.2% and benzoyl peroxide 2.5% for the once-daily treatment of moderate to severe acne vulgaris: Assessment of efficacy and safety in 2813 patients. *Journal of the American Academy of Dermatology* 59, 792–800. doi:10.1016/j.jaad.2008.06.040.

Tirado‐Sánchez, A., Espíndola, Y. S., Ponce‐Olivera, R. M., and Bonifaz, A. (2013). Efficacy and safety of adapalene gel 0.1% and 0.3% and tretinoin gel 0.05% for acne vulgaris: results of a single-center, randomized, double-blinded, placebo-controlled clinical trial on Mexican patients (skin type III–IV). *Journal of Cosmetic Dermatology* 12, 103–107. doi:10.1111/jocd.12031.

Valkenhoef, G. van (2018). *GeMTC R package: model generation for network meta-analysis: gertvv/gemtc*. Available at: https://github.com/gertvv/gemtc [Accessed January 29, 2019].

van Valkenhoef, G., Dias, S., Ades, A. E., and Welton, N. J. (2016). Automated generation of node-splitting models for assessment of inconsistency in network meta-analysis. *Research Synthesis Methods* 7, 80–93. doi:10.1002/jrsm.1167.

Viechtbauer, W. (2019). *metafor: Meta-Analysis Package for R*. Available at: https://CRAN.R-project.org/package=metafor [Accessed May 30, 2019].

Wiegell, S. R., and Wulf, H. C. (2006). Photodynamic therapy of acne vulgaris using methyl aminolaevulinate: a blinded, randomized, controlled trial. *Br. J. Dermatol.* 154, 969–976. doi:10.1111/j.1365-2133.2005.07107.x.

Xu, J. H., Lu, Q. J., Huang, J. H., Hao, F., Sun, Q. N., Fang, H., et al. (2016). A multicentre, randomized, single-blind comparison of topical clindamycin 1%/benzoyl peroxide 5% once-daily gel versus clindamycin 1% twice-daily gel in the treatment of mild to moderate acne vulgaris in Chinese patients. *J Eur Acad Dermatol Venereol* 30, 1176–1182. doi:10.1111/jdv.13622.

Xu, X., Zheng, Y., Zhao, Z., Zhang, X., Liu, P., and Li, C. (2017). Efficacy of photodynamic therapy combined with minocycline for treatment of moderate to severe facial acne vulgaris and influence on quality of life. *Medicine (Baltimore)* 96, e9366. doi:10.1097/MD.0000000000009366.

Yin, R., Hao, F., Deng, J., Yang, X. C., and Yan, H. (2010). Investigation of optimal aminolaevulinic acid concentration applied in topical aminolaevulinic acid-photodynamic therapy for treatment of moderate to severe acne: a pilot study in Chinese subjects. *Br. J. Dermatol.* 163, 1064–1071. doi:10.1111/j.1365-2133.2010.09860.x.

Zhang, J. Z., Li, L. F., Tu, Y. T., and Zheng, J. (2004). A successful maintenance approach in inflammatory acne with adapalene gel 0.1% after an initial treatment in combination with clindamycin topical solution 1% or after monotherapy with clindamycin topical solution 1%. *J Dermatolog Treat* 15, 372–378. doi:10.1080/09546630410021702.
